# Getting to the roots of it: Genetic and hormonal control of root architecture

**DOI:** 10.3389/fpls.2013.00186

**Published:** 2013-06-18

**Authors:** Janelle K. H. Jung, Susan McCouch

**Affiliations:** Department of Plant Breeding and Genetics, Cornell UniversityIthaca, NY, USA

**Keywords:** root growth, root development, hormone interactions, root system architecture, genetics, rice, *O. sativa*

## Abstract

Root system architecture (RSA) – the spatial configuration of a root system – is an important developmental and agronomic trait, with implications for overall plant architecture, growth rate and yield, abiotic stress resistance, nutrient uptake, and developmental plasticity in response to environmental changes. Root architecture is modulated by intrinsic, hormone-mediated pathways, intersecting with pathways that perceive and respond to external, environmental signals. The recent development of several non-invasive 2D and 3D root imaging systems has enhanced our ability to accurately observe and quantify architectural traits on complex whole-root systems. Coupled with the powerful marker-based genotyping and sequencing platforms currently available, these root phenotyping technologies lend themselves to large-scale genome-wide association studies, and can speed the identification and characterization of the genes and pathways involved in root system development. This capability provides the foundation for examining the contribution of root architectural traits to the performance of crop varieties in diverse environments. This review focuses on our current understanding of the genes and pathways involved in determining RSA in response to both intrinsic and extrinsic (environmental) response pathways, and provides a brief overview of the latest root system phenotyping technologies and their potential impact on elucidating the genetic control of root development in plants.

## INTRODUCTION

The exploration of root biology lags far behind above-ground vegetative and reproductive growth and development in plants. There is a vast array of studies on root biology, but the literature is dispersed, highly fragmented, and difficult to search because there are no comprehensive phenotypic databases for plants. Many studies of root genes have been classified based on discovery technique [i.e., mutant, quantitative trait loci (QTL), transgenic analyses] or response variable (hormones, microbial populations, insects, nutrients, water levels), but they have not been joined into a systemic understanding of root genetics. Furthermore, comprehensive ontology terms pertaining to root biology have yet to be established, let alone adopted, and gene functional annotation linking phenotypic characteristics into mechanistic pathways and networks is incomplete. Recently, genome-wide association study (GWAS) approaches both advance and demand better integration of genetic studies, annotations, and pathways into a more complete and searchable data network.

Effective GWAS requires the efficient integration of genotyping, phenotyping, and informatics capabilities. The continued development of increasingly rapid, low-cost, high-throughput genotyping and sequencing technologies, such as second and third generation sequencing and high density single-nucleotide polymorphism (SNP) arrays, have made it straightforward for researchers to generate massive amounts of genotypic data on individuals and populations of interest. The speed, efficiency, and cost of high-throughput precision phenotyping of those same populations lags far behind, requiring significant investments of money, time, and labor to generate the data needed for large-scale mapping studies. The selection of traits measured may be limited due to a lack of quantitative measurement resolution and/or accuracy, leading to the frequent description of traits in qualitative classes that combine multiple biological processes, as opposed to specific quantifiable traits that each measure a distinct biological step or the result of a particular process. Furthermore, existing database resources that seek to compile and integrate phenotypic and physiological data with genotypic data, such as the Database of Genotypes and Phenotypes (dbGaP; http://www.ncbi.nlm.nih.gov/sites/entrez?db=gap) and PhenomicDB (Groth et al., 2010), are limited by low data submission and limited curation capacity. While these databases are emerging as useful resources for human and bacterial data, plant-related datasets, particularly in relation to root system biology, are still woefully underrepresented.

Lack of comprehensive phenotypic and informatics resources is currently one of the most limiting factors for leveraging the power of GWAS. Although much about gene function, expression, and pathway or network interaction remains to be discovered, the plant genetics community has accumulated phenotypic data from both field and controlled environments during the last half-century. If properly structured and organized, these data could be interrogated to assist with candidate gene identification and interpretation of GWAS output. The problem is that there is no efficient way to access, parse, and cross-reference these data and therefore, they remain fragmented, dispersed and incompletely indexed. Because the collection, curation, and biological application of phenomic data is much more complicated and multi-dimensional than genotypic data, it has yet to be standardized and streamlined into automated processing modules. As a result, finding, integrating and interrogating the components of complex phenotypes, particularly those associated with plant root system architecture (RSA), requires the intervention of expert biologists who manually search through the literature to discover relevant QTLs, pathways and candidate genes. The annotation process is a complex, multi-step, iterative adventure for the scientist interested in defining relevant genes and networks for association or linkage mapping analyses.

This review was motivated by the need to identify *a priori* candidate genes involved in rice RSA, morphology, growth, and development related to the interpretation of an association mapping study based on a rice diversity panel that had been genotyped with 700,000 SNPs and screened for 19 components of seedling 3D RSA [unpublished data, McCouch and Kochian labs, Cornell University and United States Department of Agriculture-Agricultural Research Service (USDA-ARS)]. We identified from the literature known genes involved in RSA, which encompasses a range of heterogenous traits involved in many different aspects of plant growth architecture, morphology and phenology. After narrowing the search space using GWAS, we integrated positional information about candidate genes found through mutant analysis, orthologous gene identification, comparative mapping, trait similarity, pathway, and network extension, with the our candidate gene regions identified by GWAS. This was aided by the use of ontology and synteny-related informatics to find genes underlying GWAS peaks and QTLs (Lawrence and Harper, 2008; Vilella et al., 2009; Chen et al., 2012; Lamesch et al., 2012). This article provides a comprehensive review of the genetics underlying root growth, development, and response to environmental stimuli. We provide tables of genes that have been associated experimentally and *in silico* by sequence homology with root development in rice, along with positional information and gene ontology (GO) evidence codes to facilitate database population and curation (Tables S2 and S3 in Supplementary Material).

## DEFINING ROOT SYSTEM ARCHITECTURE

Root system architecture is a complex trait and refers to the spatial configuration of the root system in terms of the precise geometric arrangement of all root axes as laid down in the rooting medium. Root architecture is comprised of a whole system set of descriptors, and as such is senior to and distinct from, though naturally dependent on, the secondary fields of root anatomy, morphology, topology, and distribution; however, individual root architecture components may draw on or overlap with these fields. To clarify, root anatomy refers to the internal cellular structure and arrangement of a root; root morphology, the surface features, including diameter, root hair and cap characteristics, and contorsion; root topology, the hierarchical description of the connection of root axes to one another; and root distribution, the presence and distribution of roots in a positional gradient or grid along a horizontal and/or vertical axis.

As proposed by Fitter (1991), there are five main components of root architecture, each of which may be comprised of several specific traits or parameters. These components are: (1) branch magnitude – the number of interior links (internode segments between two branching points or nodes) or exterior links (internode segments between a branching point and an endpoint, i.e., root apical meristem (RAM); (2) topology, the pattern of branch distribution, which is usually herringbone (alternate lateral branching off a parent root), dichotomous (opposite, bifurcating branches), or radial (whorls of branches around a parent root (Hochholdinger, 2009; Lynch and Brown, 2012); (3) link/internode lengths, the distance between branch points among different root orders of an individual root, which may be averaged across a system; (4) root angles, specifically the azimuth (radial angle) of a lateral root’s (LR) emergence around the circumference of a parent root, the branching angle or departure rate of a LR from a parent root, and the spreading angle of the entire system; and (5) link radius, the diameter of any given root (Fitter, 1991).

## PATHWAYS AND NETWORKS INFLUENCING ROOT ARCHITECTURE TRAITS

As with any phenotypic manifestation, all of these simple root architecture components: branch number, branching pattern, length, orientation, angle, and diameter are developmentally controlled by complex interacting genetic pathways, which also modulate growth and developmental responses in response to the perception of environmental cues. Malamy and Ryan (2001) refer to these familiar factors – genetics, environment, and the interaction between the two – as belonging to either “intrinsic pathways” or extrinsic “environmental response pathways.”

Hormones, their receptors, signaling components, and transcription factors (TFs) make up the main chemical and molecular components of the intrinsic pathways. Extrinsic response pathways involve similar networks of receptors for environmental stimuli and their downstream signal transduction and TFs. Many components of the environmental perception and response networks are shared with or interregulated by intrinsic response pathways, and are also mediated by hormonal regulation in order to effect a growth response to external signals (see **Table 1** for a review of the major hormones and their role in modulating root architectural traits; **Table 2** for a review of the major extrinsic factors, their effects on root growth and development, and the major genes and hormones involved, and Table S1 in Supplementary Material for the key genes involved in root growth and development covered in this review). Recent studies have also identified micro-interfering RNAs (miRNAs) and small-interfering RNAs (siRNAs) which affect RSA by the post-transcriptional regulation of components involved in root growth and environmental perception and response and are themselves transcriptionally interregulated by feedback loops within the same intrinsic and extrinsic pathways (see reviews in Meng et al., 2010; Khan et al., 2011).

**Table 1:** Hormones and their involvement in root growth and development.

Hormone	Chemical compounds	Function	Hormone source	Species	Reference
Auxin	IAA	Promotes lateral root initiation by specifying lateral root founder cells	Endogenous, root tip	*At*	Casimiro et al. (2001), De Smet et al. (2007), Dubrovsky et al. (2008)
	IAA	Promotes lateral root emergence	Endogenous, shoot	*At*	Bhalerao et al. (2002)
	NAA	Increases lateral root primordia initiation and outgrowth	Exogenous	*Os, Nt*	Campanoni and Nick (2005), Sreevidya et al. (2010)
	2,4-D	Increases lateral root primordia initiation through cell division (but does not promote cell elongation and root outgrowth)	Exogenous	*Os, Nt*	Campanoni and Nick (2005), Sreevidya et al. (2010)
	IAA	Promotes primary root elongation by facilitating the response of root cells to GA3	Exogenous	*At*	Fu and Harberd (2003)
Cytokinins	Kinetin, BAP	Inhibits lateral root primordia formation by perturbing PIN gene expression and disrupting formation of a RAM auxin gradient controlling cell division to maintain the QC and neighboring initials	Increased endogenous	*At*	Laplaze et al. (2007), Dello Ioio et al. (2008); reviewed in Péret et al. (2009b)
	Kinetin, trans-zeatin	Stimulates lateral root elongation	Exogenous	*Os*	Rani Debi et al. (2005), Laplaze et al. (2007), Dello Ioio et al. (2008), reviewed in Bishopp et al. (2009)
	Kinetin, trans-zeatin	Stimulates crown root primordia formation	Exogenous	*Os*	Rani Debi et al. (2005), Hirose et al. (2007), Zhao et al. (2009)
	Zeatins, other endogenous cytokinins	Inhibits primary root elongation by reducing cell division in RAM, thus regulating RAM size	Increased endogenous	*At*	Kuderová et al. (2008), Ruzicka et al. (2009)
Gibberellins	GA3	Interacts with ethylene to promote crown root primordia outgrowth and elongation	Exogenous	*Os*	Steffens et al. (2006)
	GA3	Promotes primary root elongation in the presence of auxin by repressing growth-repressing DELLA proteins	Decreased endogenous and increased exogenous	*At*	Fu and Harberd (2003)
	GA3	Inhibits lateral root primordia initiation	Exogenous	*Pt*	Gou et al. (2010)
Ethylene	Ethylene	Promotes crown root formation at submerged nodes	Internode	*Os*	Lorbiecke and Sauter (1999)
	Ethylene	Promotes crown root emergence at submerged nodes through induction of epidermal cell death over sites of lateral root primordia formation	Internode	*Os*	Mergemann and Sauter (2000)
	Ethylene
Jasmonates	MeJA	Promotes lateral root formation through interaction with auxin pathway	Increased endogenous	*At, Gm*	Xue and Zhang (2007), Sun et al. (2009)
	MeJA	Inhibits primary root growth	Increased endogenous	*Gm*	Xue and Zhang (2007)
Abscisic acid	ABA	Induces lateral root primordia formation under non-stress conditions by modulating the auxin response	Endogenous	*At*	Brady et al. (2003)
	ABA	Maintains primary root elongation under drought stress	Endogenous	*Zm*	Saab et al. (1990)
	ABA	Inhibits lateral root outgrowth prior to lateral root meristem formation under non-stress conditions	Exogenous	*At*	De Smet et al. (2006)
Brassinosteroids	BL	May induce lateral root initiation in the presence of auxin, through modulating auxin signaling	Exogenous	*At*	Bao et al. (2004)
	BL	Induces primary root elongation in the presence of exogenous auxin (IAA) by affecting ethylene biosynthesis and the gravitropic response	Exogenous	*Zm, At*	Chang et al. (2004), Kim et al. (2007), Yun et al. (2009)
	HBR	Induces primary and crown root elongation possibly through modulating auxin signaling	Exogenous	*Hv*	Kartal et al. (2009)
Strigolactone	GR24 (synthetic strigolactone analog)	May either inhibit primary root elongation in low concentrations, or stimulate primary root growth in high concentrations, in the presence of auxin, by putative regulation of auxin efflux carriers	Exogenous	*At*	Koltai et al. (2010), Kapulnik et al. (2011), Ruyter-Spira et al. (2011)
	GR24 (synthetic strigolactone analog)	Induces primary root curving in high concentrations, in the presence of no-low auxin by inducing asymmetric cell elongation	Exogenous	*At*	Koltai et al. (2010)
	SLs	Promote crown root elongation by inducing meristematic cell division, possibly through the modulation of local auxin concentrations that regulate meristem cell number	Endogenous	*Os*	Arite et al. (2012)
	SLs	Putatively modulates auxin sensitivity by downregulating auxin efflux carrier expression to inhibit lateral root formation under low auxin levels by reducing auxin accumulation in roots, or inducing lateral root formation under high auxin concentrations by allowing optimal auxin levels to be met	Endogenous	*At*	Kapulnik et al. (2011), Ruyter-Spira et al. (2011)

*2,4-D, 2,4-dichlorophenoxyacetic acid; ABA, abscisic acid; At, *Arabidopsis thaliana*; BAP, 6-benzylaminopurine; BL, brassinolide; GA3, gibberellic acid; Gm, *Glycine max*; HBL, homobrassinolide; Hv, *Hordeum vulgare*; IAA, indole-3-acetic acid; MeJA, methyl jasmonate; NAA, 1-naphthaleneacetic acid; Nt, *Nicotiana tabacum*; Os, *Oryza sativa*; Pt, *Populus tremulus*; QC, quiescent center; RAM, root apical meristem; SLs, endogenous strigolactones; Zm, *Zea mays.**

**Table 2:** Effects of extrinsic factors in modulating root system architecture.

Factor	Condition	General effect on root growth	Genes with known involvement	Interactions with hormone pathways	Reference
**Environmental stimuli**
Gravity	Normal	Growth toward the gravity vector	*ARG1* and *2*, *PIN3* (At)	Auxin	Boonsirichai et al. (2003), Harrison and Masson (2008a),b
Light (direct root exposure)	Presence	Negative growth to blue light; positive to red/far red light	*PHOT1*, *NPH1*, *PhyA*, *PhyB* (At)	Auxin, JA	Huala et al. (1997), Kurata and Yamamoto (1997), Christie et al. (1998), Correll et al. (2003), Kiss et al. (2003), Galen et al. (2007), Costigan et al. (2011)
Water/oxygen	Root system submergence/hypoxia	CR primordia development and outgrowth (deep-water rice)	*SUB1* (Os)	GA, ethylene	Xu et al. (2006), Fukao et al. (2011)
	Drought	Mixed. General decreased LR and PR growth and LR emergence, but ABA has been shown to stimulate PR elongation and LR emergence in response to drought	*LACS2* (At); *SUB1* (Os)**	ABA	Sharp et al. (1988), Deak and Malamy (2005), De Smet et al. (2006), Macgregor et al. (2008), Wiegers et al. (2009)
**Soil nutrients**
Nitrogen	High nitrate availability	Inhibition of LR outgrowth, development and elongation	*AtNRT1.1*, *ANR1*, *AtOCT1* (At)	Auxin, ABA	Zhang and Forde (1998), Liu et al. (1999), Zhang et al. (1999), Guo et al. (2002), Munos et al. (2004), Lelandais-Briere et al. (2007), Krouk et al. (2010)
	Low nitrate availability	Localized stimulation of LR growth, branching in high inorganic N soil patches	*AtNRT1.1*, *AtNRT2.1*, *ANR1*, *AtOCT1* (At)	Auxin	Zhang and Forde (1998), Zhang et al. (1999), Malamy and Ryan (2001), Lelandais-Briere et al. (2007), Krouk et al. (2010)
Phosphorus	High phosphate availability	PR growth promoted, LR growth prohibited	*PDR2*, *LPR1*, *WRKY75* (At)**	Auxin	Linkohr et al. (2002); Ticconi et al. (2004), Ticconi et al. (2009), Shane and Lambers (2005), Reymond et al. (2006), Schulze et al. (2006), Devaiah and Raghothama (2007), Devaiah et al. (2007), Perez-Torres et al. (2008)
Phosphorus (con’t)	Low phosphate availability	Root foraging: increased LR initiation, outgrowth, forming a shallow, highly branched system	*PDR2*, *LPR1*, *PHR1*, *AtSIZ1*, *PHO2* (At), *OsPTF1* (Os), *PHI2* (Nt)**	Auxin, CK, ethylene, GA, SLs	Schmidt and Schikora (2001), Williamson et al. (2001), Franco-Zorrilla et al. (2002), López-Bucio et al. (2002), Sano and Nagata (2002), Ma et al. (2003), Shimizu et al. (2004), Ticconi et al. (2004), Ticconi et al. (2009), Miura et al. (2005), Shane and Lambers (2005), Yi et al. (2005), Bari et al. (2006), Reymond et al. (2006), Schulze et al. (2006), Devaiah and Raghothama (2007), Devaiah et al. (2007), Jiang et al. (2007), Perez-Torres et al. (2008)
Sulfur	High sulfate availability/sufficiency	Not highly studied	*SULTR1;2*, *SLIM1* (At)**	Auxin, JA, CK	Ohkama et al. (2002), Hirai et al. (2003), Maruyama-Nakashita et al. (2003), Nikiforova et al. (2003), Buchner et al. (2004), Hoefgen and Nikiforova (2008), Lewandowska and Sirko (2008), Takahashi (2010)
	Low sulfate availability	Mixed. Short-term sulfur limitation proposed to stimulate LR growth with longer-term deficiency causing overall decreased growth	*SULTR1;1*, *SULTR1;2*, *SLIM*, *OAS*, *NIT3*, *BIG*, *IAAs* (At)**	Auxin, JA, CK	Leustek et al. (2000), Saito (2000), Takahashi et al. (2000), Kutz et al. (2002), Ohkama et al. (2002), Yoshimoto et al. (2002), Maruyama-Nakashita et al. (2003), Maruyama-Nakashita et al. (2004), Nikiforova et al. (2003), Buchner et al. (2004), Bouranis et al. (2008), Hoefgen and Nikiforova (2008), Lewandowska and Sirko (2008)
**Phytotoxins**
Aluminum	High Al^3+^	Inhibition of LR initiation and outgrowth, swollen, malformed root tips	*ETR1*, *EIN2*, *AtACSs*, *AtACOs*, *AtPIN*, *AUX1*, *PME*, *AtCHIA*, *CALS*, ALS1 (At); *EXPA10*, *STAR1* and *2*, *ART1* (Os)	Auxin, ethylene	Foy (1984), Delhaize et al. (1993), Kochian (1995), Alonso et al. (1999), Matsumoto (2000), Lee and Kende (2002), Tsuchisaka and Theologis (2004), Yokoyama and Nishitani (2004), Eticha et al. (2005), O’Malley et al. (2005), Jones et al. (2006), Sivaguru et al. (2006), Larsen et al., 2007, Sun et al. (2010)
Sodium chloride	High salinity	Mixed. General decrease in root growth due to slower epidermal cell division and elongation	*HKTs*, *GLRs*, *NSCCs*, *CNGCs*, *SOS1–3*, *NX1* (At)	Auxin, ABA, CK, ethylene, GA	Kuiper et al. (1990), Zidan et al. (1990), Liu and Zhu (1998), Apse et al. (1999), Gaxiola et al. (1999), Leng et al. (2002), Quintero et al. (2002), Tester and Davenport (2003), He et al. (2005), Mahajan and Tuteja (2005), Khadri et al. (2006), Cao et al. (2008), Bano (2010)
**Symbioses**
Root nodulation	Pre-symbiosis Nod factor-induced	None known
	High colonization	Nodule formation, putative suppression of LR emergence	*Nodulins*, *LHK1* (Lj), *MtCRE*, *ARR*, *NSP1* and *2*, *NIN*, *ENOD11*, *ERFs* (Mt)	Auxin, ABA, BRs, CK, ethylene, GA, SA	Nutman (1948), Nap and Bisseling (1990), Verma et al. (1992), Catoira (2000), Journet et al. (2001), Borisov et al. (2003), Charron et al. (2004), Lohar et al. (2004), Kalo et al. (2005), Smit et al. (2005), Gonzalez-Rizzo et al. (2006), Marsh et al. (2007), Middleton et al. (2007), Frugier et al. (2008), Vernie et al. (2008), Ding and Oldroyd (2009), Markmann and Parniske (2009), Ferguson et al. (2010)
Arbuscular mycorrhizal	Pre-symbiosis Myc-factor-induced	LR elongation	*DMI1* and *2*, *MtENOD11* (Mt), *OsPOLLUX2*, *OsCCAMK2*, *CYCLOPS1* (Os, Mt, Lj)**	Auxin, ABA, CK, SLs	Endre et al. (2002), Stracke et al. (2002), Kosuta et al. (2003), Olah et al. (2005), Hogg et al. (2006), Gutjahr et al. (2009); Messinese et al., 2007; Yano et al., 2008; Horváth et al. (2011)
	High AM colonization	Variable increases in root mass, thickness, length, and LR number dependant on host species	*LRT1* (Zm)	Auxin, ABA, CK, ethylene	Hetrick et al. (1988), Berta et al. (1990), Berta et al. (1995), Berta et al. (2002), Dixon (1990), Hetrick (1991), Barker and Tagu (2000), Paszkowski and Boller (2002), Vierheilig et al. (2002), Fitze et al. (2005), Olah et al. (2005), Ludwig-Muller and Guther (2007), Parniske (2008), Gutjahr et al. (2009)

*At, *Arabidopsis thaliana*; BRs, brassinosteroids; CR/CRP, crown root/crown root primordia; Lj, *Lotus japonicus*; LR/LRP, lateral root/lateral root primordia; Nt, *Nicotiana tabacum*; Os, *Oryza sativa*; PR, primary root; Ps, *Pisum sativum*; QC, quiescent center; RAM, root apical meristem; SA, salicylic acid; SLs, strigolactones; Zm, *Zea mays.**

To date, the vast majority of research elucidating the genes and pathways involved in root architecture development has been done with the simple, dicot taproot system of *Arabidopsis thaliana* (Scheres et al., 1996; Ueda et al., 2005; Péret et al., 2009a). This has allowed for the gradual application of this knowledge in discerning conserved developmental pathways shared with monocot crown root (CR) systems, primarily studied in cereal crops such as rice (*Oryza sativa* L.) and maize (*Zea mays* L.).

## THE IMPORTANCE OF ROOT ARCHITECTURE

The 3D configuration of a root system is important mechanically, providing physical anchorage of the plant in soil, and physiologically, in nutrient and water sensing and uptake, and in response to soil biota. The rate of root system growth and its vertical and horizontal spread can affect seedling vigor, neighbor competition, and exploitation of different limiting resources, such as phosphorus, nitrogen, and water, through root growth or support of symbioses, and can be highly specific to environmental conditions – a root architecture which may favor the growth of a plant under low water conditions, may impede its growth in flooded soil. The specific growth and development characteristics of a plant’s root system also confers some degree of developmental plasticity to the organism in dealing with nutrient and water availability, seasonal and climate changes, beneficial or disease causing organisms, or toxic compounds in soil. Together, these qualities of anchorage, soil nutrient exploitation, and developmental plasticity as determined by root architecture can have far-reaching effects on maximal yield, especially under stress, and yield stability, and a greater understanding of the genes and pathways involved in root architectural development may be translated into the breeding of improved crop varieties.

## INTRINSIC PATHWAYS – GENETIC AND HORMONAL REGULATION OF ROOT ARCHITECTURE

### PRIMARY ROOT INITIATION, DEVELOPMENT, AND ELONGATION

**FIGURE 1:**
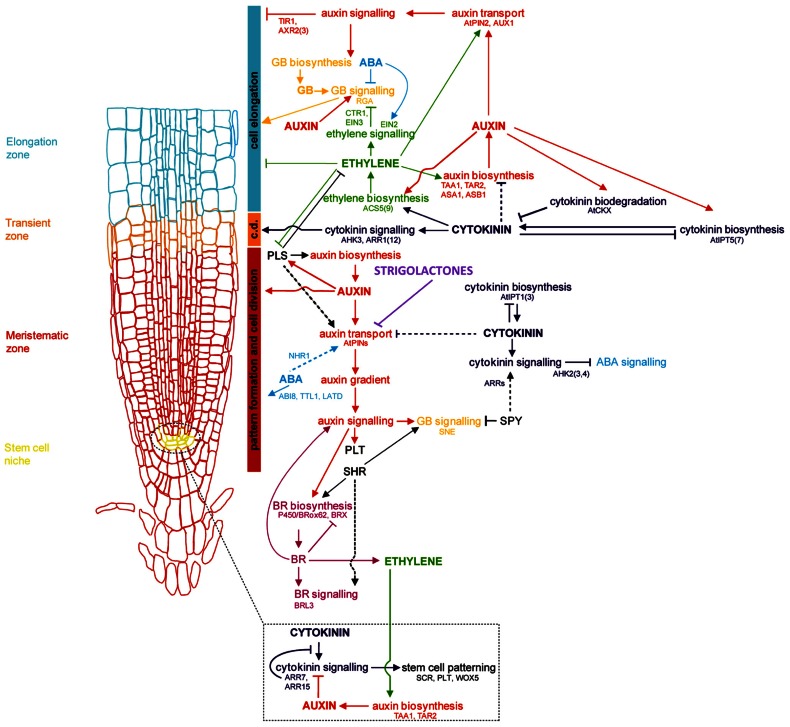
**Genetic and hormonal control of primary root development in *Arabidopsis* Model of the current understanding of hormone interaction and genetic regulation of primary root and general root apical meristem growth and development in *Arabidopsis***. Important genes involved in integrating signals from different hormone pathways are shown in black; hormone networks are color-coded; dashed lines represent unresolved or indirect relations. The fundamental role of auxin-mediated signaling in controlling all major aspects of root growth, from cell division, differentiation, and elongation, can be visualized, as well as the antagonistic regulation of auxin by cytokinins, and secondary regulation by other hormones, including ABA, ethylene, GA, brassinosteroids (BRs), and strigolactones. c.d. is “cell differentiation,” in reference to the transition zone where cell differentiation is initiated (modified from Benkova and Hejatko, 2009).

The primary root (PR), derived from the radicle and laid down during embryogenesis, grows to form the foundation of the dicotyledonous taproot system, and is the first root of the fibrous, CR-based root system of monocots. Establishment of the RAM of the PR involves cell identity differentiation and the formation and maintenance of a quiescent center (QC) and stem cell population. In *Arabidopsis*, auxin signaling and its antagonistic feedback by cytokinins (CKs) have been implicated in the development of a root stem cell niche ( Muller and Sheen, 2008; Kartal et al., 2009; Moubayidin et al., 2009; Pernisova et al., 2009; Ruzicka et al., 2009). The secondary regulation of auxin signaling by gibberellins, and brassinosteroids has also been implied (Sabatini et al., 1999; Frigerio et al., 2006). Polar auxin transport by the AUXIN1/LIKE AUXIN (AUX1/LAX) family of auxin influx transporters and the PIN-FORMED 3 (PIN3) and PIN7 auxin efflux transporters lead to the creation and maintenance of an auxin concentration gradient with a root tip maximum (Bennett et al., 1996; Parry et al., 2001; Kramer, 2004; Blilou et al., 2005; Carraro et al., 2006; Swarup et al., 2008; Liu et al., 2009; Wang et al., 2009; see reviews in Petrásek and Friml, 2009; Overvoorde et al., 2010; **Figure 1**). Several multidrug resistant/P-glycoprotein (MDR-PGP) subfamily members of the ATP-binding cassette subfamily B (ABCB) are also key auxin influx and efflux membrane transporters (Noh et al., 2001, 2003; Luschnig, 2002).

Strigolactones (SLs), a new class of plant hormones and rhizosphere signaling molecules have also been implicated in PR development based on crosstalk with auxin signaling. In the presence of auxin, exogenous application of the synthetic SL analog GR24 has been found to either inhibit *Arabidopsis* PR elongation in low concentrations, or stimulate PR growth in high concentrations by putative regulation of the auxin efflux carriers PIN1, PIN3, and PIN7 (Aida et al., 2002; Kapulnik et al., 2011; Ruyter-Spira et al., 2011). GR24 has also been found to induce PR curving in high concentrations, in the presence of no or low auxin levels by inducing asymmetric cell elongation (Koltai et al., 2010). It should be noted, however, that due to the increased stability of GR24 in aqueous solution, as compared with natural SLs, the effects of this synthetic strigolactone on root growth may be misrepresented (Akiyama et al., 2010).

The presence or absence of auxin transcriptionally regulates many genes involved in general root growth and development through the action of auxin/indole-acetic acid (Aux/IAA) and Auxin Response Factor (ARF) modules (De Smet et al., 2010; Goh et al., 2012b). When not bound to Aux/IAA proteins, ARFs are free to recognize and bind to auxin-responsive elements (AREs) in the promoters of target genes, activating or repressing their transcription. In the absence of auxin or under low auxin concentrations, AUX/IAA proteins, negative regulators of auxin response genes (Abel, 1994) bind with their ARFs, inactivating ARF activity. Under high auxin concentrations, AUX/IAA proteins are targeted for degradation by the SCF^*TIR*^ E3 ubiquitin ligase complex (Farrás et al., 2001; Gray et al., 2001; Reed, 2001; Gagne et al., 2002; Jebanathirajah et al., 2002; Dharmasiri et al., 2005; Kepinski and Leyser, 2005; Badescu and Napier, 2006; Maraschin et al., 2009; **Figure 1**).

Other layers of ARF regulation involve miRNAs. The miR160 family has been found to play a role in *Arabidopsis* PR and LR development through its regulation of the ARF TFs, ARF10 and ARF16, which are functionally redundant but both required for root cap cell formation and development (Wang et al., 2005). Transgenic overexpression of miR160 in rice also induced severe root cap defects, suggesting the presence of a similar regulatory pathway in monocots, although the target(s) of miR160 in rice have not yet been determined (unpublished data as cited in Meng et al., 2010). Normal root cap formation in all roots is necessary for normal root system development and impinges on multiple downstream RSA components, specifically, root elongation, LR production, and root growth angle as dictated by the gravitropic response through root tip sensing (Wang et al., 2005; Band et al., 2012).

In *Arabidopsis*, a second set of TFs: SHORTROOT (SHR) and its target, SCARECROW (SCR), both GAI, RGA, SCR (GRAS) TFs, are involved in the specification and localization of stem cells and the QC, as well as root radial patterning. They affect not only PR initiation, but also root diameter, and the regulation of cell division and differentiation necessary for downstream LR development (Di Laurenzio et al., 1996; Helariutta et al., 2000; Sabatini et al., 2003; Paquette and Benfey, 2005; Lucas et al., 2011). SCR is also suggested to have a possible role in mediating a cross-response between gibberellic acid (GA), brassinosteroid, and auxin signaling involved in stem cell maintenance (Muller and Sheen, 2008; Ruzicka et al., 2009; reviewed in Benkova and Hejatko, 2009). The maize SCR homolog, *ZmSCR*, was shown to be essential for the development of the maize radicle during the formation of the coleorhizae, the unique grass structure that sheathes and protects the PR meristem (PRM) during embryogenesis and germination (Tillich, 1977; reviewed in Hochholdinger and Zimmermann, 2008).

A third set of TFs, related to the second set, are the DELLA proteins, including the *Arabidopsis* GIBBERELLIN INSENSITIVE (GAI), REPRESSOR OF GA1 (RGA) and RGA-LIKE 1, RGA-LIKE 2, and RGA-LIKE 3 (RGL1, RGL2, and RGL3), rice SLENDER RICE (SLR), and its barley homolog, SLENDER1 (SLN1), are negative regulators of GA-mediated root growth, and appear to be negatively regulated by auxin. The ubiquitination and destruction of these DELLA TFs in the presence of auxin and GA thus allow for root cell division and elongation (Dill and Sun, 2001; Ikeda et al., 2001, 2002; Chandler et al., 2002; Fleet and Sun, 2005; Perez-Perez, 2007; **Figure 1**).

## LATERAL ROOT GROWTH – FROM PRIMORDIA INITIATION TO ELONGATION

First order (or primary) LRs are roots that branch off of the taproot or adventitious roots in dicots, and the primary seminal root or CRs in monocots. These first order laterals may be short and determinate, or they may develop higher orders of ramification (second, third, fourth-order, etc. laterals). LRs account for the majority of the root mass in most plant root systems, and perform key functions in soil exploration, nutrient and water uptake, and symbiosis development. While LR production is generally developmental, it may also be adaptive, in response to environmental influences within the rhizosphere. LRs are similar in anatomy, but usually smaller in diameter than their parent root, due to a reduced number of cortical cell layers and xylem and phloem poles (Coudert et al., 2010).

**FIGURE 2:**
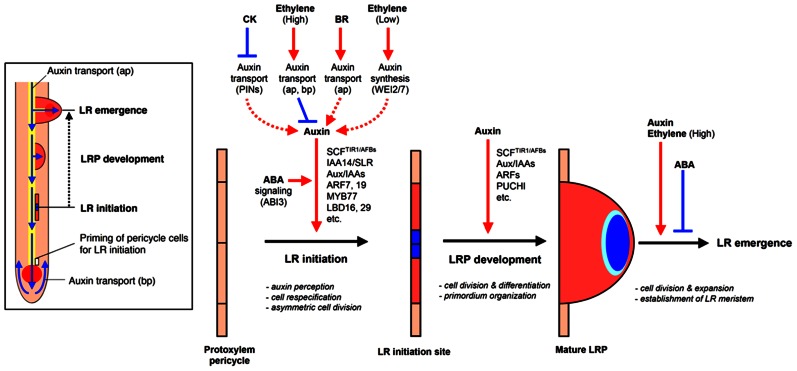
**Hormonal and genetic control of lateral root formation in *Arabidopsis***. LR formation is a three-stage process consisting of LR initiation, LRP development, and LR emergence. LR initiation is positively regulated by auxin but negatively regulated by CK and high concentrations of ethylene [high concentrations of exogenous 1-aminocyclopropane-1-carboxylic acid (ACC)]. The polar auxin transport with a balance of influx and efflux in both acropetal and basipetal directions is necessary for LR initiation and setting up auxin gradient to organize LR primordium (LRP; blue color in LR initiation site and primordium). CK inhibits auxin maxima by altering the expression of PINs, thereby inhibiting auxin gradient for LR initiation. High concentrations of ethylene or exogenous ACC, an ethylene precursor, inhibited LR initiation by enhancing acropetal (ap) and basipetal (bp) auxin transport. BR promotes LR initiation by increasing acropetal (ap) auxin transport. Low concentrations of ethylene (low concentrations of exogenous ACC) promote LR initiation by increasing Trp-dependent auxin synthesis mediated by WEI2 and WEI7. Normal ABA signaling mediated by ABI3 is necessary for proper auxin responsiveness for LR initiation. Auxin also promotes LR primordium development but CK inhibits LR primordium development and affects auxin maxima by altering the expression of PINs. ABA inhibits LR emergence whereas auxin and ethylene (via high concentrations of exogenous ACC) promotes LR emergence (modified from Fukaki and Tasaka, 2009).

Lateral root growth may be organized into four stages with different implications for RSA: (1) LR initiation, (2) LR primordia (LRP) formation, (3) LR meristem (LRM) outgrowth and emergence from the parent root, and (4) LR elongation (Malamy and Benfey, 1997, **Figure 2**). The first three stages all affect the potential number and radial orientation of LRs. Development may be halted at any stage during this process which, prior to emergence would reduce the number, position, and pattern of mature LRs; LR elongation affects LR branching angle, branch length, development rate, and whole system topology.

### LATERAL ROOT INITIATION

The first stage in LR development takes place in the parent root pericycle in *Arabidopsis*, and the pericycle and endodermis layers in crop cereals like maize and rice (Fahn, 1990; Casimiro et al., 2001). This process is characterized by founder cell identity priming and fate fixation by auxin, cell cycle activation of the founder cells, and asymmetric cell division (Malamy and Benfey, 1997; De Smet et al., 2007; reviewed in Fukaki and Tasaka, 2009). The IAA28-ARFs module, the first of three known AUX/IAA-ARF modules regulating LR development is active in this LR initiation stage for LR founder cell specification (De Rybel et al., 2010). Cell cycle reactivation and control is fundamental to LR initiation and is partially induced by the accumulation of high auxin levels in quiescent xylem pole pericycle or endodermal cells (Beeckman et al., 2001; Casimiro et al., 2001; Malamy, 2005), and the priming of specific xylem pole or endodermal cells to become LR founder cells by 15-h oscillations in the auxin level (De Smet et al., 2007). In *Arabidopsis*, this root tip-synthesized auxin gradient was found to promote asymmetric cell division of xylem pole pericycle founder cells (Casimiro et al., 2001; De Smet et al., 2007) by the auxin-induced upregulation of cell cycle genes, including cyclins and cyclin-dependent kinases (*CDK*s; Soni, 1995; Meijer and Murray, 2000; Boniotti and Gutierrez, 2001), and the synchronous downregulation of CDK repressors, such as *KRP1* and *KRP2*, which inhibit the G1 to S transition phase in LRP (Himanen et al., 2002; reviewed in Fukaki et al., 2007; **Figure 2**).

Further research has suggested that cyclic changes in auxin concentration are insufficient as the sole trigger of LR initiation, and that molecular clock-coordinated oscillating gene expression within the so-called “oscillation zone,” a region encompassing the PR basal meristem and elongation zone, is also necessary for the spatial and temporal definition of LR pre-branching sites. These pre-branching sites develop LRP, but may not always grow out into fully emerged LRs (Moreno-Risueno et al., 2010). In *Arabidopsis*, two sets of 2084 and 1409 genes were found to oscillate either in phase or in antiphase, respectively, with specific waves of each phase being associated with increased expression of particular genes, mostly notably members of the ARF, NAC, myeloblastosis (MYB), and SOMBRERO TF families. T-DNA insertions in several of these genes also showed defects in LR pre-branching site initiation and reduced LR number (Moreno-Risueno et al., 2010).

## LATERAL ROOT PRIMORDIA FORMATION

The formation of LRP is characterized by several rounds of anticlinal and periclinal cell division (Malamy and Benfey, 1997). As modeled in *Arabidopsis*, this process generates a patterned LRP similar to the PR tip (DiDonato et al., 2004). Mutant and transgenic studies in *Arabidopsis* suggest that the formation of both the LRM and the PRM are driven by equivalent, if not the same, hormonal and genetic factors (Malamy and Benfey, 1997). Auxin is the primary signaling hormone regulating LRP development through the formation of an auxin gradient (Péret et al., 2009a). This gradient is modulated upstream by low levels of antagonistic CKs which would otherwise repress LRP formation via the disruption of auxin efflux PIN protein localization, which itself is partly responsible for creating the auxin gradient (Laplaze et al., 2007). CK specifically affects the rate of cell differentiation between the cell division and elongation/differentiation zones but does not affect the rate of cell division in the meristem (Dello Ioio et al., 2008). SLs may also regulate LRP formation, possibly by altering auxin sensitivity through downregulating the expression of auxin efflux carriers such as PIN1, thus inhibiting LR formation under low auxin levels by reducing auxin accumulation in roots, or inducing LR formation under high auxin concentrations by allowing optimal auxin levels to be met (Ruyter-Spira et al., 2011).

The *Arabidopsis* GTP:GDP ANTIPORTER/PROTEIN HOMODIMERIZATION (GNOM) protein also appears to play an essential role in regulating PIN protein trafficking for auxin gradient formation (Steinmann et al., 1999; Geldner et al., 2003; Laplaze et al., 2007). The accumulation of auxin in the central cells and later in the tip of the LRP signals the targeted degradation of AUX/IAA proteins, repressors of auxin-induced transcription. Furthermore, the auxin gradient enables ARF7/NPH4 and ARF19 module-upregulated transcription of target genes for cell ID and pattern formation, including other downstream TFs, such as LATERAL ORGAN BOUNDARIES DOMAIN 16/ASYMMETRIC LEAVES2-LIKE 18 (LBD16/ASL18) and LBD29/ASL16 (Okushima et al., 2005; Lee et al., 2009; Goh et al., 2012a; **Figure 2**).

### LATERAL ROOT OUTGROWTH

Lateral root primordia emergence through the overlying tissues of the parent root involves both further growth, in terms of cell elongation and division, and further differentiation, particularly the development and activation of the LRM, the definitive feature of a newly formed LR (Malamy and Benfey, 1997). Primordia emergence requires the coordinated separation of the overlying cells in the parent root in order to avoid excessive damage and infection risk (Laskowski et al., 2006; Swarup et al., 2008). In *Arabidopsis*, only three single-cell tissue layers have to be penetrated; in rice as many as 15 cell layers must be penetrated for LRP emergence (Osmont et al., 2007; Péret et al., 2009b).

This process of root cell separation for root primordial emergence is regulated by basipetal, shoot-derived auxin (Bhalerao et al., 2002) and LRP-derived auxin (Swarup et al., 2008), promoting cell separation and upregulating the expression of cell wall-remodeling genes in the endodermal, cortical, and epidermal cells layers overlaying the LRP (Swarup et al., 2008). LAX3, a high-affinity auxin influx transporter, upregulated in response to LRP-derived auxin, and specifically expressed in the epidermal and cortical cells overlaying LRP, facilitates auxin influx in these cells, spatially regulating the subsequent expression of auxin-induced genes involved in cell wall remodeling (Swarup et al., 2008). These cell wall-modification genes encode a suite of enzymes, including pectate lyases such as phospholipase A2 (PLA2), pectin methylesterases (PMEs), polygalacturonase (PG), an expansin (EXP17), and at least one known glycosyl hydrolase, GLH17, all of which are implicated in facilitating cell wall loosening and separation for LRP outgrowth to occur (Henrissat and Davies, 1997; Cosgrove, 2000; Marin-Rodriguez et al., 2002; Laskowski et al., 2006; Swarup et al., 2008; Ogawa et al., 2009; **Figure 2**).

The activation of the LRM is also thought to occur during LRP emergence from the parent root (Laskowski et al., 1995). While the genes and pathways involved in this process have yet to be elucidated, a shift in auxin signaling or source of synthesis from the parent root to the new LRM is implicated, as the arrested post-emergence growth of the *Arabidopsis* *aberrant lateral root formation3* (*alf3*) mutant can be rescued with the application of exogenous auxin, suggesting that the ability of the new LR to synthesize its own auxin may coincide or cause lateral meristem (LM) activation (Celenza et al., 1995; Péret et al., 2009b; **Figure 2**). Multiple Aux/IAA–ARF modules, including the SHY2/IAA3–ARF module (Goh et al., 2012b), may play a role in the complex networks regulating LRP development and LR emergence. These networks may also be mediated post-transcriptionally by the downregulation of LR emergence through the auxin-induced expression of *miRNA164a* and *miR164b* which target for degradation the mRNAs of NAM/ATAF/CUC1 (NAC1; Guo et al., 2005), a TF involved in transmitting auxin signals for LR emergence (Xie, 2000). Preliminary research shows this miR164-NAC1 regulatory module may also be conserved in tomato (Zeng et al., 2010) and rice (Meng et al., 2010).

### LATERAL ROOT ELONGATION

The genetic control of post-emergence LR elongation affects the rate and angle of LR growth, LRM determinancy and branching potential, all of which are important considerations in RSA. Not much is known about the genetic control of these traits; however, these are areas under active research. The *Arabidopsis* *PLETHORA1* and *2* (*PLT1* and *2*) and *CLAVATA3* (CLV3) genes are implicated in both primary and LRM maintenance of the root stem cell niche and QC, as mutants of these genes fail to maintain the QC and root stem cells, and thus stop root elongation (Aida et al., 2004; Fiers et al., 2004). *In vitro* application of the artificially synthesized, mature CLV3 peptide, a 12-amino acid ligand, processed from the conserved 14-amino acid CLE (CLV3/ESR) domain of a larger peptide (Fiers et al., 2006), and peptide synthesis or overexpression of other members of its greater CLE family of related proteins sharing the conserved and essential CLE motif, all caused the termination of root development (Strabala and O’Donnell, 2006; Kinoshita et al., 2007), suggesting other CLE genes could be involved in regulating RAM identity (reviewed in Miwa et al., 2009). Cell division and elongation, particularly elongation or expansion is one of the primary drivers of root growth rate, and while the genes involved have not yet been cloned, the maize mutants *short lateral root1* and *2* (*slr1* and *slr2*) display short, slow-growing LRs on their primary and embryonic CRs, which microscopy studies haves attributed to a decrease in cell elongation (Hochholdinger et al., 2001). Hormonal interactions also play a role in LR growth: auxins, ethylene, and abscisic acid (ABA) have been shown to inhibit LR elongation, while CKs promote elongation (Rani Debi et al., 2005; Iwama et al., 2007; **Figure 2**, **Table 1**). Amongst the many auxin transporters potentially involved in LR elongation, ABCB19/MDR1, an important shoot basipetal auxin transporter, has also been shown to be important for root acropetal auxin transport and necessary for maintenance of a high enough auxin concentration to support post-emergence LR elongation at a normal rate (Wu et al., 2007).

The angle of LR growth is thought to be at least partially under genetic control due to tropic responses, as different *Arabidopsis* and rice accessions display variations in LR angle (Mullen and Hangarter, 2003; Iyer-Pascuzzi et al., 2010), which may be attributable to differences in intrinsically programed LR gravitropic setpoint angle (GSA), the angle of growth relative to the gravity vector (Digby and Firn, 2002). Mutant analyses of *Arabidopsis* lines with a normal PR gravitropic response, but variations in LR GSA suggest that the genetic control of GSA may be independent between LR and PR, and that GSA may be mediated by auxin signaling and a root phototropic response (Mullen and Hangarter, 2003). 

## CROWN ROOTS – FROM INITIATION TO ELONGATION

Crown roots, also called nodal or shoot-borne roots, are adventitious roots unique to monocots and part of normal monocot root system development. Along with their associated LRs, CRs make up the bulk of the fibrous monocot root system. CRs may be developmentally separated into two different types: the embryonic CRs – seminal roots which form around the coleoptilar node along with the PR (radicle) during embryogenesis, and the post-embryonic CRs that arise during germination and throughout the life of the plant (Hochholdinger and Tuberosa, 2009). Along with dicot root and the monocot seminal PR, all CRs, both embryonic and post-embryonic, can be considered primary order roots, as like the radicle they arise from the main stem of the plant and not from another root as do LRs.

### CROWN ROOT PRIMORDIA INITIATION AND DEVELOPMENT

Most root development research has focused on PR and LR, thus much if the current knowledge about the genetic control of CR development is deduced from studies of maize and rice mutants or based on comparative analysis with *Arabidopsis* PR, LR, and adventitious root studies. The overarching hormonal regulation and the gene families regulating PR, LR, CR (in monocots), and adventitious (in dicots) root growth appear to be largely conserved (Hochholdinger et al., 2004; Coudert et al., 2010). The functions of individual genes in the genetic pathways regulating the development may, however, be slightly different.

Crown root primordia (CRP) initials are produced from periclinal divisions of parenchyma cells which give rise to the pattern arrangement of differentiated epidermis/endodermis initials, central cylinder cells, and root cap initial cells (Itoh et al., 2005). This is followed by the establishment of epidermis and endodermis by periclinal divisions of the endodermis–endodermis initials, and then the formation of the cortical cells and central metaxylem (Itoh et al., 2005).

Similar to early processes in PR and LR development, the initiation and development of CRs is also controlled by auxin-mediated signaling (reviewed in Rebouillat et al., 2009). OsGNOM1, an ortholog of *Arabidopsis* GNOM1, was found to be involved in regulating proper PIN1 auxin efflux protein trafficking, and thus the polar auxin transport necessary for auxin gradient formation to signal the proper asymmetrical division of parenchyma cells for CRP development (Geldner et al., 2003; Liu et al., 2009; Péret et al., 2009b; Richter et al., 2010). Maize and rice homologs of the *Arabidopsis* *SHR* and *SCR* genes, GRAS TFs, also have been shown to be essential for the radial patterning necessary for CRP development. With a similar endogenous expression pattern to the *Arabidopsis* genes and *in vitro* evidence of the capacity for interaction between each species pair, it is likely that in monocots the two TFs share a similar role in CR, as opposed to LRP development and interact with each other to restrict the formation of the endodermis to a single-cell layer (Cui et al., 2007).

**FIGURE 3:**
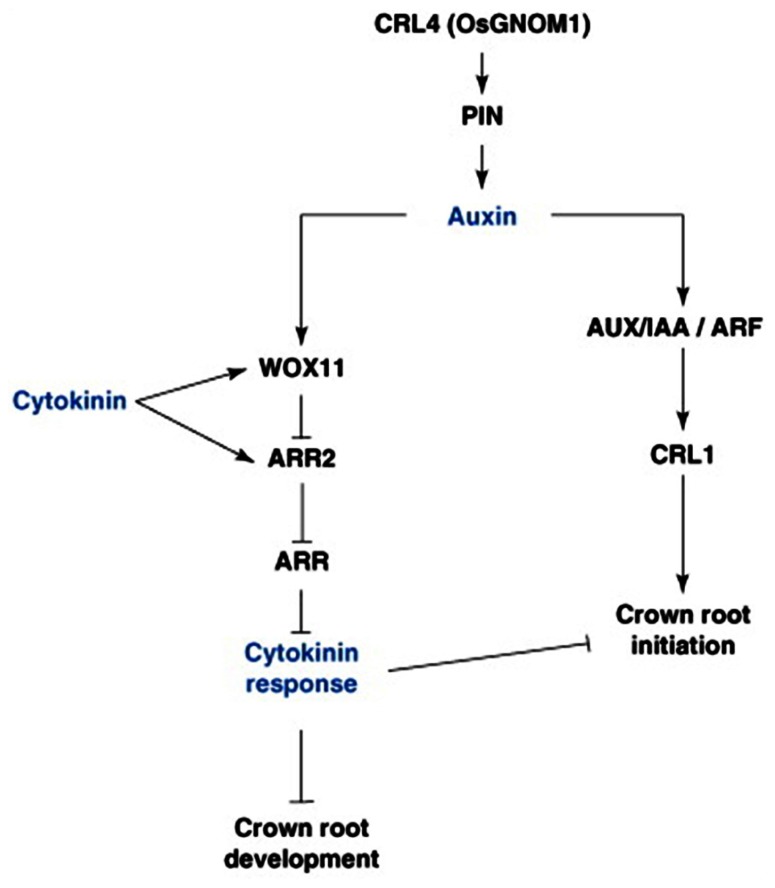
**Hormonal and genetic control of crown root formation in rice**. Crown root initiation in rice is promoted by auxin, and regulated by the inhibitory influence of cytokinin. Arrows represent the positive regulatory action of one element of the network on another one. A line ending with a bar represents the negative regulatory action of one element of the network on another one. ARF, Auxin Response Factor; ARL, ARR, type-A RESPONSE REGULATOR; AUX/IAA, AUXIN/INDOLE-3-ACETIC ACID; CRL4, CROWN ROOTLESS4; GNOM1, GTP:GDP ANTIPORTER/PROTEIN HOMODIMERIZATION1; PIN1, PIN-FORMED1; WOX11, WUSHEL-Related Homeobox 1 (Coudert et al., 2010).

There is also evidence to suggest that the monocot radicle/primary seminal root, the embryonic CRs, and the post-embryonic CRs may be under different genetic control. The monogenic maize mutant *rootless concerning crown and seminal roots* (*rtcs*) does not form any CRs, just the PR and its associated laterals (Hetz et al., 1996). Other monogenic maize mutants display less severe root developmental phenotypes: *lateral rootless 1* (*lrt1*) does not develop CRs at the coleoptilar node or any LRs on the PR or remaining embryonic CRs (Hochholdinger and Feix, 1998), whereas the *rum1* mutant has no embryonic CRs, and few, late-developing LRs and post-embryonic CRs (Woll et al., 2005). Rice mutants *crown rootless1* (*crl1*) and *adventitious rootless1* (*arl1*), found to be allelic, have no CRs or CRP, fewer LRs off the PR, and an abnormal gravitropic response (Inukai et al., 2001). Rice *ARL1/CRL1* and *RTCS* have been shown to encode LBD (Lateral organ Boundary Domain) proteins similar to those encoded by the *Arabidopsis* *LBD16* and *29* genes (Inukai et al., 2005; Liu et al., 2005; Taramino et al., 2007). All genes are members of the same family and are probably auxin responsive, having AREs; however, they each have different functions. *LBD16* and *29* are involved in LR formation in *Arabidopsis*, the maize *RTCS* gene is involved only in CR development, and the rice *ARL1/CRL1* gene in both LR and CR development (**Figure 3**; Inukai et al., 2005; Liu et al., 2005; Taramino et al., 2007).

Similar to LR formation in *Arabidopsis*, CKs also plays a secondary role in mediating CR development in monocots through antagonism of auxin-related signaling pathways. The rice *WUSCHEL-RELATED HOMEOBOX11* (*WOX11*) gene encodes an auxin and CK-induced TF expressed in early CRP and the actively dividing regions of the shoot apical meristem (Zhao et al., 2009) and found to repress the CK-upregulated type-A response regulator gene, *RR2* (Jain et al., 2006), which may function as a negative regulator of CK signaling and may repress cell proliferation in the CR meristem, thus repressing CR emergence (Zhao et al., 2009). Knockout mutants of *WOX11* exhibited inhibited CR growth, while overexpression of the gene increased rates of CR cell division, leading to precocious CR growth. Additionally both mutant and overexpressor lines also showed altered transcription of auxin and CK-responsive genes, suggesting that *WOX11* may play a pivotal role in integrating auxin and CK signaling to control cell division rates in the CRP (**Figure 3**; Zhao et al., 2009).

### CROWN ROOT OUTGROWTH AND ELONGATION

While the formation of CRP is under genetic and physiological control, the emergence of developing CRs from stem nodes is at least partially influenced by the environment. Mergemann and Sauter (2000) found that in accessions of deep-water rice, the buildup of ethylene caused by submergence induces the death of epidermal cells above CRP, thus promoting emergence of CRs through the epidermis of the submerged nodal branches.

Recent studies on this phenomenon have shown that GA is also involved as a non-essential but synergistic upregulator of CRP emergence and elongation rate in the presence of ethylene, and ABA as a likely inhibitor of both ethylene and GA signaling pathways (Steffens and Sauter, 2005; Steffens et al., 2006). While the specific hormone biosynthesis, signaling, and target genes implicated in this H_2_O_2_ programed cell death pathway have not yet been identified, it has been shown that the epidermal cells overlying CRP may be predestined to die, exhibiting a lower transcription level of *METALLOTHIONEIiN2b* (*MT2b*), which encodes a reactive oxygen scavenger that, in higher levels, would prevent cell damage by H_2_O_2_ (Steffens and Sauter, 2009). It is possible that CRP emergence may also be auxin-regulated, as rice RNAi-knockdown lines of the *OsPIN1* gene, which encodes an auxin efflux carrier, show arrested CRP emergence (Xu et al., 2005); however, the physiological mechanism by which auxin signaling influences CRP emergence is yet unknown.

Strigolactones may play a role in positively regulating CR elongation through promoting root meristematic cell division (Arite et al., 2012), potentially through modulating auxin flux. Rice *dwarf* mutants for genes involved in SL biosynthesis (SL-deficient rice mutants *max3/rms5/d17*, *max4/rms1/d10*, and *d27*) or SL signaling (SL-insensitive rice mutants *max2/rms4/d3* and *d14*) were found to have a short CR phenotype due to an apparent decrease in cell division, leading to a narrower meristematic zone (Arite et al., 2012). This decreased cell division may be due to SL-modulation of local auxin levels, affecting meristem cell number as seen in PRs of homologous *Arabidopsis* SL-deficient and SL-insensitive mutants (Kapulnik et al., 2011; Ruyter-Spira et al., 2011); however, the specific mechanism of SL effect on root growth has yet to be fully elucidated.

## EXTRINSIC PATHWAYS – ROOT SYSTEM ARCHITECTURE CHANGES IN RESPONSES TO ENVIRONMENTAL STIMULI

**FIGURE 4:**
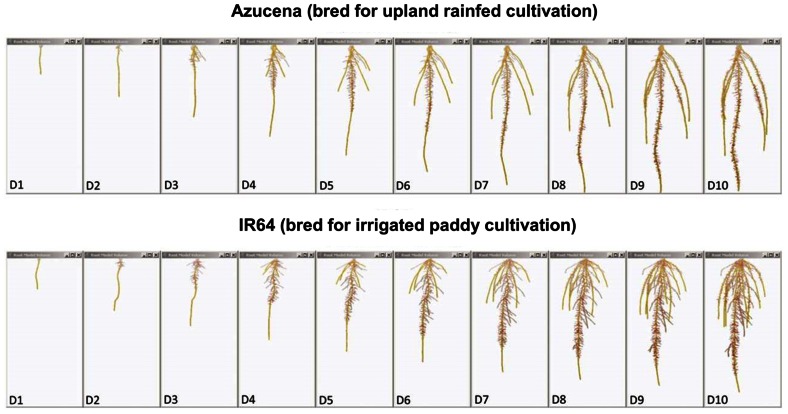
**Root system models of two rice varieties bred for contrasting agricultural systems**. These root system models, generated from image series of seedling rice root systems of cv. Azucena (top), and cv. IR64 (bottom) over 10 days of growth (D1–D10) in a clear, gellan-gum nutrient media show how the breeding of crop varieties adapted to particular cultivation systems and agroecological environments has resulted in inadvertent selection for different crop root architectures. Azucena, a rice variety bred for an upland rainfed growing environment develops a deeply rooted primary and crown root architecture consistent with rapid growth in search of water, whereas IR64, bred for a water-sufficient, irrigated paddy system is more shallowly rooted, but develops longer, highly branched lateral roots in the top part of the root system to scavenge nutrient resources, such as nitrogen and phosphorus, from near the soil surface. Primary and crown roots are shown in yellow; the root system skeleton is shown in red (modified from Clark et al., 2011; models were generated using RootReader3D software).

The intrinsic genetic pathways detailed previously control the normal development of plant root systems by directing the primordia initiation, outgrowth, and elongation of various root types. Modulation of these pathways in response to the environment allow plants the phenotypic plasticity to modify specific components of their RSA to exploit limiting nutrient resources and respond to a constantly fluctuating complex of biotic and abiotic stresses. Even different ecotypes or varieties from the same species that are adapted for growth in dissimilar rhizosphere environments can vary widely in intrinsic root system development schemes and plasticity responses, resulting in heritably different RSAs (Malamy, 2005; Suralta et al., 2008; Clark et al., 2011; Gowda et al., 2011; Pacheco-Villalobos and Hardtke, 2012; **Figure 4**). While the genes and pathways involved in environmental perception and signaling may be unique to a particular stimulus, root growth response pathways often feed into the underlying genetic pathways by co-opting hormonal regulation. Current understanding of the genetic and hormonal regulation of RSA changes induced by tropisms, nutrient availability, toxic compounds, symbioses, and abiotic stresses are reviewed here and in **Table 2**.

### GRAVITY

The downward growth of roots influences RSA traits such as root angle, length, and depth, is primarily controlled by a positive gravitropic response, hypothesized to be perceived through the sedimentation of statoliths (amyloplasts – starch-containing plastids, or other plastids, such as chloroplasts) within statocytes, specialized gravity-sensing cells in the root tip (reviewed in Morita, 2010). The mechanism of gravity sensing is yet unknown, but is postulated to be through statolith pressure or movement receptor perception, or pressure-based opening of mechanosensitive ion channels (reviewed in Perrin et al., 2005). In *Arabidopsis*, ALTERED RESPONSE TO GRAVITY1 (ARG1) and ALTERED RESPONSE TO GRAVITY-LIKE2 (ARL2), J-domain proteins localized to endomembrane organelles, are thought to intact with one another to form a gravity signal transduction complex, promoting rapid, transient cytoplasmic alkalinization through Ca^2+^ influx, and the redistribution of auxin efflux carrier PIN3 to the lower membrane of the statocytes (Boonsirichai et al., 2003; Harrison and Masson, 2008a,b). The relocalization of PIN3 results in the asymmetric redistribution of auxin along the new, lowest side of the root tip (Young and Evans, 1996; Lucas et al., 2008; Nishimura et al., 2009), followed by basipetal auxin transport to the root distal elongation zone, mediated by the auxin influx carrier AUX1 and efflux carrier ETHYLENE INSENSITIVE ROOT1 (EIR1; Blancaflor and Masson, 2003; Swarup et al., 2005; Band et al., 2012; Brunoud et al., 2012). This new auxin gradient is thought to signal the upregulation of genes leading to cell elongation along the top end of the distal elongation zone, thus producing root tip curvature downward (Blancaflor and Masson, 2003; reviewed in Petrásek and Friml, 2009). In addition to auxin, other phytohormones or signaling molecules, including CKs (Aloni et al., 2006), reactive oxygen species (Cervantes, 2001; Joo et al., 2001), flavonoids and ethylene (Buer et al., 2006; Edelmann and Roth, 2006) may be involved in gravitropic root tip curvature growth response by controlling differential cell elongation in parallel with auxin or as regulators of the auxin-mediated signaling pathway. 

The aforementioned concept of a genetically controlled measure of gravitropism, the GSA – the equilibrium angle (or range of angles) from vertical at which an organ shows no gravity-induced differential growth (Digby and Firn, 1995), has bearing on RSA traits such as CR and LR angle of growth. Mutant analyses of *Arabidopsis* lines with a normal PR gravitropic response but variations in LR GSA suggest that the genetic control of GSA may be independent between LR and PR, and that GSA may be mediated by auxin signaling and a root phototropic response (Mullen and Hangarter, 2003).

### LIGHT

Although the root systems of most plants are largely underground and not exposed to light, plant roots may be exposed to light through ambient diffusion or soil upheaval and have been found to possess phytochromes, phototropins, and cryptochromes, including both red and blue light photoreceptors (Ruppel et al., 2001; Mullen et al., 2002; Galen et al., 2007; Molas and Kiss, 2008). Root responses to light have been studied mostly in *Arabidopsis*, which is found to display a negative phototropic response to blue light, mediated by the root phototropin (PHOT1; Huala et al., 1997; Christie et al., 1998; Galen et al., 2007), and a positive phototropic response to red light, mediated by the root-expressed phytochromes A and B (PhyA and PhyB; Kiss et al., 2003). PhyA also promotes root elongation under exposure to far red (Kurata and Yamamoto, 1997; Correll et al., 2003; Costigan et al., 2011) and blue light (Costigan et al., 2011). Auxin concentration differentials may be partially responsible for root growth responses to shoot light exposure, as the proper plasma membrane localization of the auxin efflux transporter PIN2 was found to be greatly increased in light-grown, but the protein was targeted for vesicular degradation in dark-grown seedlings (Laxmi et al., 2008). Jasmonic acid (JA) is also implicated in a root-localized light response, as one study has demonstrated that phytochromes, or more specifically, phytochrome chromophores are necessary for the JA-mediated root growth inhibition (Costigan et al., 2011).

### WATER AVAILABILITY

Given that one of the main functions of the root system is water uptake, soil water availability and soil hydraulic conductivity, especially in the extreme conditions of drought leading to water deficiency or flooding leading to soil saturation and hypoxia, is arguably the most important environmental factor influencing root growth and development. Studies in *Arabidopsis* have shown that decreasing osmotic potential as a representation of drought stress reduces the LR outgrowth and emergence from LRP of plants grown on agar plates (Deak and Malamy, 2005). Similar research in maize has shown that small increases in negative water potential stimulate PR elongation, but further water stress decreases the rate of PR growth (Sharp et al., 1988; Wiegers et al., 2009).

Hormonal signaling controlling root growth responses to water availability is not yet fully elucidated, however, ABA has been shown to stimulate PR elongation and LR emergence in response to drought (De Smet et al., 2006). In contrast, in flooded deep-water rice plants, a decreased internode ABA level and the concurrent accumulation of GA and the ethylene produced as a response to hypoxia and flooding stress, initiates the programed cell death of adventitious root primordia epidermal cells, allowing the adventitious root development and outgrowth (Mergemann and Sauter, 2000; Steffens et al., 2006). Similarly, the *Arabidopsis* *LONG CHAIN FATTY ACID SYNTHETASE2* (*LACS2*) gene essential for cutin biosynthesis was shown to be required in order for plants to be able to synthesize a cutin layer that suppresses LR emergence under low water availability (Macgregor et al., 2008). The rice ethylene response factor (ERF)-like TF SUBMERGENCE1 (SUB1; Xu et al., 2006), a TF involved in mediating responses to both plant submergence and drought, may also be one of many genes involved in regulating root growth under water stress, as osmotic stress-induced inhibition of root growth was found to be slightly suppressed in rice varieties with a functional copy of the SUB1 gene (Fukao et al., 2011).

## GROWTH IN RESPONSE TO SOIL NUTRIENTS

Plant root adaptive growth in response to soil macro and micronutrients depends on a wide array of variables: nutrient forms, availability, concentration, localization, and nutrient behavior in soil, as well as the nutrient status of the plant. Similar to the tropic responses above, plant root growth in response to a nutrient stimulus requires four main steps: stimulus perception, signal transduction, target gene regulation, gene product mediation of growth response.

### NITROGEN

Nitrogen, the most limiting nutrient to plant growth is an interesting example of these highly plastic plant responses to nutrient availability, as it can inhibit LR outgrowth, development and elongation under high N conditions, or in soil with low inorganic nitrogen, soil patches with high inorganic nitrogen can have a local, stimulatory effect on LR elongation and branching within the high N area. *Arabidopsis* senses nitrate through the PR tip, with downstream components of the nitrate LR growth response pathway include high and low-affinity *Arabidopsis* *NITRATE TRANSPORTERS1.1* and *2.1* (*AtNRT1.1* and *2.1*; Zhang et al., 1999; Malamy and Ryan, 2001), and nitrate-responsive TFs, including the MADS box TF *ARABIDOPSIS NITRATE REGULATED1* (*ANR1*; Zhang and Forde, 1998).

The nitrate transporters may be either nitrate sensors or, transporters that facilitate N movement for detection via another protein. AtNRT2.1 is necessary for LR growth repression in plants with a high external carbon to nitrogen value (Malamy and Ryan, 2001; Little et al., 2005; Remans et al., 2006b), and AtNRT1.1 is a dual-affinity transporter induced by both auxin and nitrate and important for nitrate uptake under high N conditions (Liu et al., 1999; Guo et al., 2002; Munos et al., 2004). AtNRT1.1 is also an auxin influx facilitator, decreasing its auxin transport activity in response to nitrate sensing, and is proposed to repress LR development by promoting basipetal auxin transport out of LRP under low external nitrate conditions (Krouk et al., 2010). ANR1 mediates the localized N response, regulating the increased proliferation of LRs in N-dense patches, and may be a direct or indirect target of the signal perception/transduction pathway involving AtNRT1.1 (Zhang and Forde, 1998; Remans et al., 2006a). ABA may also act in the same pathway as nitrate by inhibiting LR growth under high N conditions (Signora et al., 2001; De Smet et al., 2003). SLs appear to be upregulated in plants under low N conditions (Yoneyama et al., 2007b); however, whether increased these SL levels have a definite impact on root growth has yet to be determined.

Changes in RSA may also be induced depending on the prevailing available organic form of nitrogen, such as L-glutamate or carnitine. In *Arabidopsis* seedlings, the sensing of L-glutamate by the PR tip inhibits cell division in the PRM and induces LR formation and outgrowth. L-glutamate may act more as a signaling molecule as opposed to a nitrogen source, as several *Arabidopsis* auxin-signaling mutants display varying levels of sensitivity to L-glutamate (Walch-Liu et al., 2006), and a rice glutamate receptor mutant displays a host of RSA changes, with short PR and LR, reduced cell division and RAM cell death (Li et al., 2006). Carnitine, transported in *Arabidopsis* by AtOCT1, has been shown to stimulate LR formation, perhaps by locally affecting the C:N ratio important in modulating LR development (Lelandais-Briere et al., 2007).

### PHOSPHORUS

Phosphorus is the second most limiting nutrient because of its high affinity to bind metals in acidic and alkaline topsoil layers, forming insoluble substrates. Phosphorus is taken up by plants as phosphate (Pi), either directly by the root system or, in arbuscular mycorrhizae hostplants, may also be transferred through the fungal symbiont – the genetic control of which will be explored in detail later in this paper.

Under high Pi conditions in *Arabidopsis*, PR growth is promoted, while LR growth is inhibited (Linkohr et al., 2002). Under natural conditions where Pi is limiting, plants adopt a root foraging strategy to explore topsoil layers for phosphorus. This Pi foraging strategy may be accomplished through one of several different RSA and physiological changes. In *Arabidopsis* and rice, growth shifts to favor an increased root:shoot ratio, with a higher initiation and outgrowth of LRs, forming a shallow, highly branched root system (Williamson et al., 2001; López-Bucio et al., 2002; Shimizu et al., 2004). Under low Pi conditions, *Arabidopsis* PR growth is inhibited (Williamson et al., 2001; Linkohr et al., 2002; López-Bucio et al., 2002), while root hairs increase in density and length (Bates and Lynch, 1996, 2000). In legumes, including soybean, pea, and common bean (*Phaseolus vulgaris*), basal root growth angle is shifted from a downward to a more horizontal direction (Bonser et al., 1996), though a recent study shows the opposite effect in *Arabidopsis*, with LR GSAs shifting to a steeper, downward orientation under low Pi conditions (Bai et al., 2013). Several different families of plants develop proteoid or cluster roots – highly branched bunches of LRs just below the soil surface that secrete phosphatases and organic acids which solubilize bound phosphate for uptake (Shane and Lambers, 2005; Schulze et al., 2006).

In *Arabidopsis*, the PR tip is the key organ involved in phosphate sensing, and the initial effect of low external Pi perception is the inhibition of PR growth by the loss of meristem activity and cell elongation (Williamson et al., 2001; Sanchez-Calderon et al., 2005). While a plant Pi-receptor has yet to be identified, studies suggest that the P_5_ type ATPase PHOSPHATE DEFICIENCY RESPONSE2 (PDR2), and multicopper oxidase LOW PHOSPHATE ROOT1 (LPR1) function in an endoplasmic reticulum-localized Pi-signaling pathway (Ticconi et al., 2004, 2009; Reymond et al., 2006). PHOSPHATE STARVATION RESPONSE1 (PHR1; Bari et al., 2006), an *Arabidopsis* MYB-like TF that binds the promoter sequences of low Pi-induced genes, and its regulator SMALL UBIQUITIN-LIKE MODIFIER1 (AtSIZ1; Miura et al., 2005), a small ubiquitin modified E3 ligase, and the downstream PHOSPHATE2 (PHO2), an E2 conjugase, and the microRNA *miR-399*, which regulates PHO2 expression, are all involved in Pi-deficiency-related transcriptional changes (Bates and Lynch, 2000; Bari et al., 2006). The *Arabidopsis* WRKY75 TF is also induced during Pi-deprivation and may modulate both phosphate and non-phosphate induced LR development and control the transcription of genes such as high-affinity Pi transporters important for Pi uptake (Devaiah and Raghothama, 2007; Devaiah et al., 2007). The Pi-induced tobacco bZIP TF PHOSPHATE INDUCED2 (PHI2; Sano and Nagata, 2002) and rice bHLH TF PI STARVATION-INDUCED TRANSCRIPTION FACTOR1 (OsPTF1; Yi et al., 2005) may also have a role in modulating low Pi-induced changes in RSA.

Increased auxin sensitivity, decreased CK sensitivity, and changes in auxin transport and localization appear to be at least partially responsible for Pi stress-induced LR development. A shift in auxin overaccumulation from the PR apex to the LRP, or an increased sensitivity of LRP to auxin have been suggested as proposed mechanisms for increases in LRP emergence and LR density (Franco-Zorrilla et al., 2002; López-Bucio et al., 2005; Nacry et al., 2005). TIR1 auxin receptor-dependent degradation of TF-repressing AUX/IAA proteins is essential for LR development in Pi-stressed seedlings (Perez-Torres et al., 2008). The effect of auxin under low Pi conditions is also regulated by CK signaling, which represses auxin-induced gene transcription Pi-starved *Arabidopsis* plants display a decreased response to CK, partly due to the reduced expression of the CR receptor *CRE1* (Franco-Zorrilla et al., 2002). Ethylene perception is likely also necessary for increased root hair development and LR elongation and decreased PR elongation under low Pi conditions (Schmidt and Schikora, 2001; López-Bucio et al., 2002; Ma et al., 2003) and has additionally been shown to affect Pi stress-induced changes in basal root growth angle in bean (Lynch and Brown, 2001). Similar to CK, GA acts as a negative repressor of Pi-induced root architecture changes under low Pi conditions; Pi-deficient plants accumulate DELLA proteins, which repress GA-induced root growth suppression and thus allow for auxin-mediated LR initiation and elongation (Jiang et al., 2007). SL production is induced by low Pi in many species including tomato, *Arabidopsis*, pea, and rice (López-Ráez and Bouwmeester, 2008; Umehara et al., 2010; Kohlen et al., 2011; Foo et al., 2012; Mayzlish-Gati et al., 2012). Some studies suggest that increased production and exudation of SLs under soil Pi or N deficiency is dependent on whether the plant (1) is an arbuscular mycorrhizal fungi (AMF)-compatible host, and (2) whether it is dependent on the arbuscular mycorrhizal symbiosis (AMS) for Pi and N uptake (Yoneyama et al., 2007a,b, 2008; Umehara et al., 2010); however, what effect, if any, this increased SL exudation has on root growth is unclear. Exogenously applied GR24 appears to increase LR formation under low Pi or decrease LR formation under sufficient Pi though the F-box protein MORE AXILLARY GROWTH2 (MAX2), a putative component of the SL-signaling pathway (Kapulnik et al., 2011; Ruyter-Spira et al., 2011).

### SULFUR

Sulfur, taken up by plant roots as sulfate, is another limiting plant macronutrient, and is essential for the synthesis of methionine and cysteine. Sulfur deficiency can have significant effects on RSA; sulfate limited *Arabidopsis* and maize plants increase their LR production, developing an extensive, highly branched root system, often at the expense of shoot growth (Kutz et al., 2002; Bouranis et al., 2008). Another conflicting *Arabidopsis* study found a decrease in LRP and emerged LR under low-sulfate growth conditions (Dan et al., 2007). To rectify these two opposing developmental outcomes, a two-state model was proposed wherein short-term sulfur limitation let to increased LR growth for sulfate foraging, but longer-term sulfate deficiency led to overall decreased growth and photosynthesis, ending in premature senescence (Hoefgen and Nikiforova, 2008; Lewandowska and Sirko, 2008).

While the genes involved in internal and external sulfate sensing and transcriptional regulation have not yet been cloned and characterized, several components of root sulfate import and signal transduction have been identified. Of the five major classes of sulfate transporters identified in *Arabidopsis* and rice (Takahashi et al., 1999; Buchner et al., 2004; reviewed in Takahashi, 2010), the group 1 high-affinity transporters are essential for root sulfate uptake. *Arabidopsis* *SULFATE TRANSPORTER1;2* (*SULTR1;2*) is expressed under both sulfate-sufficient and low-sulfate conditions and transcriptionally regulated by the ETHYLENE-INSENSITIVE3-LIKE3 TF SLIM1, whereas the *SULTR1;1* gene induced only under sulfate stress (Takahashi et al., 2000; Yoshimoto et al., 2002; Maruyama-Nakashita et al., 2004) and upregulated by *O*-acetylserine (thiol) lyase (OAS), a rate-limiting enzyme involved in sulfate assimilation into cysteine (Leustek et al., 2000; Saito, 2000). 

Auxin may play a central role in LR production under sulfate stress. In *Arabidopsis*, sulfate deficiency activates the transcription of *NITRILASE3* (*NIT3*), which converts indole-3-acetonitrile to the auxin IAA (Kutz et al., 2002). However, while NIT3 activity is especially upregulated in LRP under sulfate limitation, increased concentrations of auxin have not been proven (Kutz et al., 2002; Lewandowska and Sirko, 2008). Studies of sulfur-limitation regulated auxin signaling genes such as *BIG*, named for the huge 560 kD calossin-like protein it encodes, required for the polar transport of auxin (Gil et al., 2001), as well as the auxin TF genes *IAA13*, *IAA28*, and *ARF-2*, indicate that auxin is likely involved in the indirect regulation of sulfur homeostasis and short to long-term sulfur deficiency responses (Hirai et al., 2003; Maruyama-Nakashita et al., 2003; Nikiforova et al., 2003; Hoefgen and Nikiforova, 2008; Lewandowska and Sirko, 2008). JA may also play a role in sulfur regulation, as demonstrated by research in *Arabidopsis* finding low sulfur JA biosynthesis genes upregulated under low sulfur in (Maruyama-Nakashita et al., 2003), exogenous application of JA promoted sulfur assimilation and there is also evidence to suggest that CKs and sucrose may affect sulfur responsive gene transcription (Ohkama et al., 2002).

## TOXIC COMPOUNDS

High soil concentrations of naturally occurring soluble salts, aluminum, and heavy metals, such as cadmium, lead, and chromium, can be highly phytotoxic and seriously impair plant root growth. Plants exhibit two main strategies to manage toxic soil compounds: (1) producing root exudates that bind and neutralize the toxin in the rhizosphere, and (2) actively transporting the compound into the root, but neutralizing and sequestering it in vacuoles for safe accumulation, or eliminating it through exudation.

### ALUMINUM TOXICITY

Aluminum is the third most abundant element and the most abundant metal in the Earth’s crust. Aluminum toxicity is one of the major constraints to yield productivity worldwide, especially in the acid soils of the tropics and subtropics that comprise almost 50% of all non-irrigated arable land in those regions (Uexküll and Mutert, 1995). At a soil pH of 5.5 or less, Al is solubilized into Al^3+^, its phytotoxic form, which has a high plant uptake affinity through diffusion (Kochian, 1995). Al^3+^ is highly toxic to plant growth, causing a rapid inhibition of root apical cell expansion and elongation, and the eventual cessation of cell division, resulting in a stunted, brittle root system with swollen malformed tips, inhibited LR initiation and outgrowth, deformed root hairs, and a poor nutrient and water uptake capacity (Foy, 1984; Delhaize et al., 1993; Kochian, 1995; Matsumoto, 2000).

In addition to *Arabidopsis*, several cereal crops, such as, maize, rice, sorghum, and wheat have been used to examine the physiological and molecular mechanisms of aluminum tolerance, as members of the grass family appear to be among the most resistant to aluminum toxicity (Delhaize et al., 1993; Magalhaes et al., 2004; Mao et al., 2004; Caniato et al., 2007). The two most well-studied mechanisms of aluminum tolerance include external avoidance, through root secretion of organic acids such as malate, citrate, and oxalate, which chelate Al^3+^ ions in the rhizosphere, preventing their diffusion into roots (Miyasaka et al., 1991; Delhaize et al., 1993; Ma and Furukawa, 2003), and true, internal tolerance, by the uptake, organic acid chelation, and sequestration of bound aluminum substrates (Matsumoto et al., 1996; Ma et al., 2001; Huang et al., 2009b; Klug and Horst, 2010); however, only the molecular pathways involved in Al^3+^-stress-induced RSA changes will be discussed below.

The site of Al^3+^ sensitivity in maize is the root apex (Ryan et al., 1993); however, exposure of only the distal transition zone of maize roots to Al^3+^ was found to reduce cell elongation in the elongation zone (Sivaguru and Horst, 1998), suggesting the presence of a diffusible signal between the zones, later found to be the ethylene-mediated basipetal transport of auxin (Kollmeier et al., 2000; Sun et al., 2010). In *Arabidopsis*, the ethylene receptor gene *ETHYLENE RECEPTOR1* (*ETR1I*; O’Malley et al., 2005) and the ethylene signal transducer *ETHYLENE INSENSITIVE2* (*EIN2*; Alonso et al., 1999) were found to be necessary to the Al^3+^ induced inhibition of root elongation (Sun et al., 2010). These genes, likely along with other members of the ethylene signaling pathway, are essential for Al^3+^ induced upregulation of the *Arabidopsis* ethylene synthesis genes *1-AMINOCYCLOPROPANE-1-CARBOXYLIC ACID SYNTHASE2*, *6*, and *8* (*AtACS2*, *6*, *8*) and *1-AMINOCYCLOPROPANE-1-CARBOXYLIC ACID OXIDASE1* and *2* (*AtACO1*, and *2*; Tsuchisaka and Theologis, 2004), followed by the upregulation of auxin transporters *AtPIN2* and *AUX1*, leading to auxin distribution changes that are likely responsible for the inhibition of root elongation (Sun et al., 2010).

The binding of Al^3+^ to negative binding sites on root cell walls and plasma membranes, has also been proposed to inhibit root elongation by increasing wall and membrane rigidity leading to transverse ruptures between the dermal and outer cortical cell layers from the inner cortex, and causing root tip damage (Kopittke et al., 2008), as well as impaired membrane function (Miyasaka et al., 1989; Ahn et al., 2001; Jones et al., 2006; Sun et al., 2010). Al^3+^ binds primarily to negatively charged pectin in cell walls; the degree of binding has been found to be determined not by the amount of pectin, but by its negative charge as modulated by methylation (Eticha et al., 2005) by PME (Schmohl et al., 2000).

Interestingly enough, the blocking of Al^3+^ cell wall binding sites (Huang et al., 2009a) may be a major mechanism of aluminum resistance in rice, which does not appear to secrete enough chelating organic acids to rely on an Al^3+^ external avoidance strategy (Ma et al., 2002). Two genes, *SENSITIVE TO ALUMINUM RHIZOTOXICITY1* and *2* (*STAR1* and *2*) encode the nuclear binding domain and transmembrane domain, respectively, of an ABC transporter with specificity for uridine diphosphate (UDP) glucose that is upregulated following root exposure to Al^3+^ (Huang et al., 2009a). Both *STAR* genes are upregulated by the constitutively-expressed rice root *ALUMINUM RESISTANT TRANSCRIPTION FACTOR1* (*ART1*), which also upregulates several other genes implicated in different aluminum tolerance mechanisms (Yamaji et al., 2009). Among these are rice homologs of genes encoding proteins implicated in modulating root elongation and cell wall elasticity: namely an α-expansin EXPA10, members of which have been shown to decrease cell wall extension potential when exposed to Al^3+^ (Gao et al., 2008), and are additionally downregulated in response to Al^3+^ (Lee and Kende, 2002), and an *Arabidopsis* cell wall-associated putative endochitinase CHITINASE A (AtCHIA; Yokoyama and Nishitani, 2004), likely involved in modulating cell wall extension by regulating chitin levels (Kwon et al., 2005).

The upregulation of 1,3-β-D-glucan synthase (Bhuja et al., 2004), resulting in callose deposition in root apices, especially in endodermal and cortical cell walls (Budíková, 1999), is another signal of Al^3+^-induced injury (Jones et al., 2006; Sivaguru et al., 2006). It is proposed that this abnormal callose deposition may inhibit both symplastic and apoplastic flow (Sivaguru et al., 2000, 2006; reviewed in Horst et al., 2010), causing inhibition of root growth. It is not yet understood whether callose deposition actually represents Al^3+^-induced injury, is a secondary cell-strengthening response to aluminum damage, or possibly even a defense response to block further Al^3+^ binding.

### SALINITY

Salinity is estimated to affect at much as 20% of the world’s agricultural land and 20% of the world’s irrigated cropland (Flowers and Yeo, 1995) due to a number of natural and man-made factors, including natural salinity and coastal proximity, poor water or fertilizer management, the clearing of vegetation, and prolonged cycles of drought and flooding. In most saline soils, sodium chloride (NaCl) is the most soluble and abundant salt, with calcium and magnesium chloride in lesser concentrations. The dominant causes of plant saline toxicity are complicated by the effects of saline soils on external root osmotic stress, which affects water and nutrient uptake, especially in competition with potassium (K^+^) and calcium (Ca^2+^), and internal ionic stress most frequently from the buildup of high sodium (Na^+^) concentrations (Munns and Tester, 2008).

Different species of plants have varying levels of salt tolerance, from the highly halophilic saltbush (*Atriplex* spp.) to highly sensitive species, such as rice and *Arabidopsis* (Munns and Tester, 2008). RSA is generally not affected as severely as shoot branching and leaf expansion under salt stress; in many plants, root growth decreases under NaCl treatment due to reduced epidermal cell division and elongation rates, likely in response to the osmotic stress (Kurth et al., 1986; Zidan et al., 1990). Salt stress also was shown to increase LR production and suppress PR elongation in *Arabidopsis* (He et al., 2005), induce programed cell death in rice root tips (Li et al., 2007), as well as raise the root death rate in sensitive tomato accessions (Snapp and Shennan, 1992).

Of the many mechanisms of salt tolerance – uptake inhibition, internal sequestration, leaf exclusion, root efflux, and osmotic stress tolerance (reviewed in Munns and Tester, 2008) – root uptake inhibition, efflux, and osmotic stress tolerance have probably the greatest local effect in mediating RSA changes and root growth responses. Na^+^ is thought to enter the root by passive diffusion through either high-affinity K^+^ transporters (HKTs), such as the rice OsHKT2;1 (Horie et al., 2007), or through non-selective cation channels (NSCCs); possibly glutamate activated receptors (GLRs), which complex with glutamate to form a channel (Demidchik et al., 2010), or cyclic nucleotide-gated channels (CNGC; Leng et al., 2002; Tester and Davenport, 2003). In the current *Arabidopsis* model of Na^+^ stress signaling, internal Na^+^ presence is perceived by a yet unknown sensor, triggering cytosolic Ca^2+^ flux sensed by the Ca^2+^ sensor Salt Overly Sensitive3 (SOS3; Liu and Zhu, 1998), which complexes with and activates SOS2, CBL-interacting protein kinase (Quintero et al., 2002). The SOS2/SOS3 complex is involved in controlling three different Na^+^ transporters to maintain a low cytoplasmic [Na^+^]. These include: SOS1, a plasma membrane Na^+^/H^+^ antiporter that increases Na^+^ efflux out of the cell (Zhu et al., 1998; Quintero et al., 2002), a vacuolar Na^+^/H^+^ exchanger (NHX1), which facilitates N^+^ sequestration in vacuoles (Apse et al., 1999; Gaxiola et al., 1999) and may negatively regulate HKTs, such as *Arabidopsis* HKT1, restricting Na^+^ buildup in the cytoplasm (Uozumi et al., 2000; Rus et al., 2001; Zhu, 2002; reviewed in Mahajan and Tuteja, 2005). Ionic balance between Na^+^, H^+^, Ca^2+^, and K^+^ is essential; under low K^+^ conditions in rice, moderate levels of Na^+^ influx into the roots through OsHKT2;1 transporters were found to be beneficial in partially maintaining root elongation otherwise inhibited under low K^+^; however, the biochemical advantage to this phenomenon is not yet understood (Horie et al., 2007, 2009).

Symbiotic interaction with plant rhizobacteria and arbuscular or ectomycorrhizal fungi have also been shown to mitigate saline toxicity and alleviate salt stress, perhaps by modulation of root ion and nutrient levels (Sheng et al., 2008; Dimkpa et al., 2009; Evelin et al., 2009; Luo et al., 2009; Shilev et al., 2010). Internal fluctuations in the concentrations and transport of several hormones, including the stress-induced ABA, as well as ethylene, auxin, CKs, and possibly GAs, are observed in response to salinity stress and are mostly linked to shoot-to-root Na^+^ stress signaling (Kuiper et al., 1990; He et al., 2005; Khadri et al., 2006; Cao et al., 2008; Bano, 2010). Ethylene and auxin signaling were, however, found to be required for increased LR production in salt-stressed *Arabidopsis* seedlings in connection with the TF AtNAC2, induced by upstream EIN2 transduced ethylene signaling (He et al., 2005). Interestingly enough, auxin and ABA are also implicated in the opposite RSA response of *Medicago truncatula* under salt stress: decreased PR elongation, LRP initiation, and LR emergence. In this study, ABA and salt stress both induced upregulation of HOMEOBOX 1 (HB1), a TF found to represses LRP emergence by repressing the downstream TF LBD1, which would otherwise activate downstream genes promoting LRP outgrowth (Ariel et al., 2010). Microarray comparative analysis of rice, *Arabidopsis* and ice plant (*Mesembryanthemum crystallinum*) revealed several dozen common genes with salinity-induced transcriptional changes, including genes involved in stress perception and osmotic regulation (Pareek et al., 2007). The precise identity of root architecture-related genes regulated by salt stress-induced TFs have yet to be determined.

## SYMBIOSES

Plant root symbiotic associations with microbes, most notably the mycorrhizal and rhizobial symbioses, have long been known to promote plant nutrient uptake efficiency.

In order to support these symbioses, host plant root architecture may undergo a number of significant changes throughout the pre-contact root–microbe signaling, symbiosis development, and establishment processes detailed in the following sections on mycorrhizal and rhizobial symbioses below. Although both symbioses induce different changes in root architecture and plant nutrient status, they share some similar components in their signaling and early developmental pathways, the so-called “SYM pathway” (Parniske, 2008). Recently, a set of seven common SYM genes/proteins required for both symbioses were identified (Parniske, 2008). These include: the Leu-rich repeat receptor kinase SYMRK/DOES NOT MAKE INFECTION2 (DMI2), activated after Nod factor signal perception (Endre et al., 2002; Yoshida and Parniske, 2005); two nuclear membrane-localized cation channels, CASTOR (Imaizumi-Anraku et al., 2004) and POLLUX/DMI1 (Ané et al., 2004; Imaizumi-Anraku et al., 2004); two nucleoporins, NUP85 (Saito et al., 2007) and NUP133 (Kanamori et al., 2006), all necessary for inducing the Ca^2+^ spike signal (Kosuta et al., 2008); the calcium/calmodulin-dependent protein kinase (CCaMK; Lévy et al., 2004; Mitra et al., 2004; Tirichine et al., 2007), which acts downstream of Ca^2+^ spiking and is thought to transduce the calcium signals, partly through the physical interaction and phosphorylation of CYCLOPS, a protein with a nuclear localization signal and carboxy-terminal coiled-coil domain protein of unknown function (Yano et al., 2008; Horváth et al., 2011). Intersecting research on the arbuscular mycorrhizal (AM) and rhizobial symbioses have largely been carried out on the model legumes *Lotus japonicus* and *Medicago truncatula*, as neither *Arabidopsis*, nor any of the other non-leguminous model plants have the ability to host the rhizobial symbiosis.

## MYCORRHIZAL SYMBIOSES

Over 90% of land plants form symbioses with mycorrhizal fungi. These symbioses improve plant nutrient capture through fungal mineral scavenging and transfer to the plant, and can be linked to significant changes in plant root architecture. Most of the research in this field, and subsequently in this review, is focused on the AMS, the most common type of mycorrhizal symbiosis, found in over 80% of plant species and involving the ~200 obligate biotroph fungal species of the Glomeromycota phylum (Schüßler et al., 2001; Strack et al., 2003). The AMS has ancient origins – estimated to be 400 million years old, it is suggested to have played a major role in the early colonization of land by plants (Pirozynski and Malloch, 1975; Simon et al., 1993). The AMS is characterized by pre-contact plant–fungal signaling, fungal contact and entry of the host plant root system, and the formation of arbuscules, highly branched fungal structures within root cortical cells that are the site of nutrient (primarily P, but also N, Zn, and Fe) transfer from the fungus to the plant and carbohydrate transfer from the plant to the fungus (reviewed in Parniske, 2008).

Pre-contact signaling, development, and maturation phases of the AMS all may induce changes in RSA, however, separating these changes from those induced indirectly as a result of improvements in plant nutrient status is challenging. Previous studies have generally reported increases in root branching as a result of colonization, yet a review of these studies reveal further complicating factors: plant root systems do not respond to AM fungal colonization in the same ways. Colonization-induced root responses appear to differ depending on host plant species, types (woody vs. non-woody; monocot vs. dicot), or varieties, soil water and nutrient status, especially of P, and possibly even the species of AM fungi (Hetrick et al., 1988; Berta et al., 1990, 1995; Olah et al., 2005; Gutjahr et al., 2009; reviewed in Hetrick, 1991; Berta et al., 2002; Parniske, 2008). Strigolactone synthesis and exudation from the roots triggers AM fungal hyphal branching, a key step in root colonization (Akiyama et al., 2005); however, the direct effect of Sls on AMS-related root growth and development is unclear and highly dependent on plant Pi and N status and concentration in the rhizosphere (see prior sections on nitrogen and phosphorus).

In maize, root thickness and overall root mass, but not LR formation, are increased by AM colonization, which also partially restores the LR growth completely absent in the *lrt1* mutant, possibly indicating the involvement of auxin signaling (Paszkowski and Boller, 2002). A partial hormonal influence in AM colonization-induced RSA changes may well be possible; studies have reported altered levels of auxin (Fitze et al., 2005), ethylene (Vierheilig et al., 2002), CK (Dixon, 1990; Barker and Tagu, 2000), and ABA in colonized roots (Herrera-Medina et al., 2007), as well as specific roles for auxin, CK, and ABA in AMS development (Barker and Tagu, 2000; Fitze et al., 2005; Ludwig-Muller and Guther, 2007). In contrast with maize, in which the AMS stimulates an increase in root thickness, but not root number, AM colonization in rice was found to induce CR elongation and both fine, determinate and long, indeterminate LR number (Gutjahr et al., 2009). Interestingly enough, while AMF-exposed three monogenic essential rice SYM gene mutants, *pollux-2*, *ccamk-2*, and *cyclops-1*, did not develop colonized roots, they showed a decrease in CRs and an increase in LRs over non-AMF-mutant controls, indicating the presence of root growth pathways induced by AM fungi, but independent of the SYM pathway (Gutjahr et al., 2009).

The only definite example of AM fungi-induced RSA development is in the legume *Medicago truncatula*, where pre-fungal contact LR formation was discovered to be induced by a diffusible factor from AM fungi, the so-called “Myc” factor of AM fungi that affects plant host signaling pathways (Olah et al., 2005). Induction of LR development by this pathway requires the proper function of two SYM pathway components, DMI1 and 2 (Endre et al., 2002; Stracke et al., 2002; Hogg et al., 2006), as well as the novel MtENOD11 protein, all of which have necessary but yet undetermined roles in pre-symbiont contact AM and *Rhizobium* symbiosis signaling (Kosuta et al., 2003; Olah et al., 2005).

## *Rhizobium*–LEGUME SYMBIOSIS

The *Rhizobium*–legume symbiosis is the most prominent and well-studied of plant associations with N-fixing bacteria, and consists of a symbiotic association between the roots of legumes (Fabaceae) and root nodule-forming, N-fixing soil bacteria of the family Rhizobiaceae. Another similar, though lesser-studied, root nodule symbiosis is the actinorhizal symbiosis between plant species in three rosid orders, the Fagales, Cucurbitales, and Rosales, and N-fixing actinobacteria of the genus *Frankia* (Swensen, 1996). Host plants in both symbioses benefit by gaining an internal supply of fixed-N, as well as potential increases in resistance to some disease and abiotic stresses, while the endosymbiotic bacteria gain a protected living environment and a carbon source supplied by plant photosynthate. Similar to the AMS, the *Rhizobium*–legume symbiosis starts with pre-contact signaling between the bacteria and host plant, followed by bacterial infection of root hairs, root hair curling, infection thread and nodule development, and bacterial colonization of nodules (reviewed in Provorov, 2000).

Colonization of legume roots may affect RSA in two ways: root nodule formation and changes in PR or LR growth. The two types of symbiotic nodules – determinate and indeterminate – differ both structurally and developmentally, and are dependent on the host plant species. Cells of the tip meristem of determinate nodules fully differentiate at maturity and are not maintained resulting in spherical nodules at uniform developmental stages, whereas the tip of the meristem of indeterminate nodules is continuously active and producing new infected tissue, creating larger and longer cylindrical or bulbous nodules with different developmental zones (reviewed in Markmann and Parniske, 2009). Studies also suggest that there is a balance between LR and nodule formation, with nodule primordia initiation dependent on the suppression of LR emergence (Nutman, 1948; Lohar et al., 2004).

Given the ancient origin and near-universality of the AMS in the plant kingdom, and the familial specificity of the rhizobial symbiosis to only the Leguminosae, it has been proposed that the rhizobial symbiosis has recruited much of the key symbiotic development pathway from the AMS, then modified and evolved genes and pathways for nodulation specific functions (Markmann and Parniske, 2009). Although the functioning alleles of the seven aforementioned known genes in the shared SYM pathway are necessary for the development of both the AM and rhizobial symbioses (Kistner et al., 2005), none of these are directly involved in symbiosis-related RSA changes. Each of these seven gene products is involved in only the early stages of the SYM signal reception and transduction pathway. The downstream, symbiosis-activated genes and networks feeding into intrinsic hormone-controlled and nutrient-modulated root growth pathways are what is actually involved in regulating *Rhizobium*-induced nodulation and LR development to balance plant nitrogen fixation needs with its carbon budget.

Cytokinin accumulation in root hairs and cortical cells after *Rhizobium* inoculation has been implicated as a key differentiation signal in stimulating root nodule organogenesis in response to Nod factor signaling (Lohar et al., 2004; Ferguson et al., 2010). CK suppresses pericycle cell division for LRP initiation, promotes cortical cell division for nodule primordia formation, and stimulates the expression of early *NODULIN* (*Nod*) genes (Bauer et al., 1996; Fang and Hirsch, 1998; Svistoonoff et al., 2010), a broad array of genes found to be transcriptionally activated or upregulated during nodulation, many of which are involved in cell wall synthesis (reviewed in Nap and Bisseling, 1990; Frugier et al., 2008). The prominent role of CK presence and/or perception in nodule formation is emphasized by studies showing pseudonodule formation in both legumes and non-legumes due to exogenously applied CK (Arora et al., 1959; Rodriguez-Barrueco and De Castro, 1973; Relic et al., 1993) and a CK-like purine derivative secreted by a *Bradyrhizobium* strain that does not produce Nod factors (Giraud et al., 2007), as well as a gain-of-function mutation in a lotus histidine kinase CK receptor *lhk1* that results in *Rhizobium* and CK-independent, spontaneous root nodule formation (Tirichine et al., 2007). CK receptors implicated in nodule development in *Medicago truncatula* include MtCRE1 (Gonzalez-Rizzo et al., 2006), an ortholog of *Arabidopsis* cytokinin receptor 1/*Arabidopsis* histidine kinase 4 (AHK4; Yamada et al., 2001), and CK-response regulators similar to the *Arabidopsis* CK-response proteins ARR4–5 (Gonzalez-Rizzo et al., 2006) and ARR10–12 (Lohar et al., 2006). TFs activated downstream of CK signaling in root cortical cells include NODULATION SIGNALING PATHWAY1 and 2 (NSP1 and NSP2; Kalo et al., 2005; Smit et al., 2005) and NODULE INCEPTION (NIN; Catoira, 2000; Borisov et al., 2003; Marsh et al., 2007). All three of these TFs are essential for nodulation, and may regulate and coordinate nodule development by regulating the expression of downstream *NODULINs* – genes expressed specifically during nodulation (Nap and Bisseling, 1990; Verma et al., 1992), such as EARLY NODULIN11 (ENOD11), a putative cell wall repetitive hydroxyl-proline-rich protein (Journet et al., 2001; Charron et al., 2004).

In addition to CK, a hormone network including auxin, JA, ABA, GA, salicylic acid (SA), brassinosteroids, and ethylene are also tightly regulated during nodule organogenesis (reviewed in Ferguson et al., 2010). Auxin, brassinosteroids, and GA are reported to be positive regulators of nodule formation, while ABA, JA, and ethylene are reported to be negative regulators, possibly by their involvement in plant stress and defense response pathways (reviewed in Ding and Oldroyd, 2009). Several *Medicago truncatula* ERFs have been found to be associated with Nod factor signal transduction, including the ERF REQUIRED FOR NODULATION (ERN; Middleton et al., 2007) and ERF REQUIRED FOR NODULE DIFFERENTIATION (EFD; Vernie et al., 2008). ABA is also thought to modulate the CK response by promoting LR growth, suppressing nodule formation, and inhibiting *Rhizobium* and Nod factor-induced gene expression (Ding et al., 2008). Most studies done on hormones and nodulation to date have only involved one to two hormone classes, thus a system-wide view of the interactions and effects of the major plant hormones on nodule organogenesis regulation has yet to be assembled.

## PHENOTYPING PLATFORMS FOR FURTHER UNDERSTANDING OF ROOT ARCHITECTURE TRAITS

High power, high resolution GWAS and sequencing methods have far outpaced phenotyping methods necessary for the discovery of regions and underlying genes involved in plant growth and development (McNally et al., 2006; Yan et al., 2009; Huang et al., 2010; Tung et al., 2010; Zhang et al., 2010). Precise, single-trait elucidation and accurate, efficient measurement are an absolute requirement for the replicated phenotyping of large panels of individuals necessary to resolve trait–genotype associations using GWAS. Traditional methods used for root growth and architecture evaluation, such as field excavation, root bagging, plate culture, core sampling, and rhizotrons (reviewed in Shashidhar et al., 2012) are poorly suited for the large number of individuals required by GWAS due to a range of issues including low volume and sampling size, poor trait complexity resolution and measurement accuracy, and high labor, time, space, and material costs. However, these traditional approaches provide invaluable information about plant growth and yield under relevant field conditions and can be productively integrated with results from newer phenotyping platforms to provide a strong rationale for prioritizing future research.

A host of new, minimally intrusive, non-destructive, whole-root system growth systems and imaging platforms have now been developed that should revolutionize our ability to explore the genetic basic of RSA. Of these, hydroponics (Famoso et al., 2010) and gel (Fang et al., 2009; Iyer-Pascuzzi et al., 2010; Clark et al., 2011) growth systems are currently amongst those best suited for RSA trait measurement and analysis for their highly controlled and standardized rooting environments, ease in whole-root system visualization and adaptability for the imposition of environmental stresses and nutrient profiles. Both of these systems involve root growth in a non-natural, liquid or semi-solid rooting environment, however, they can require tailored adjustment for use with different plant species, and are somewhat spatially and thus developmentally limited to relatively simple root systems from small or young plants. X-ray computed tomography (Lontoc-Roy et al., 2006; Perret et al., 2007; Tracy et al., 2010), nuclear magnetic resonance (NMR; Menzel et al., 2007), laser (Braga et al., 2009), ground penetrating radar (GPR) and infrared (IR) and near-infrared (NIR) imaging systems (Tirlapur and Konig, 1999; Dokken and Davis, 2007) are advantageous in their ability to visualize plant root systems grown in soil or solid rooting media, but are currently limited by their small analysis volume and often low resolution and precision, as well as their cost, accessibility, and low-throughput.

With further advancements, NMR, GPR, and IR/NIR technologies have the greatest scale-up potential for the eventual non-destructive imaging and phenotyping of field-grown plant root systems. Although these current root growth systems and imaging technologies are still unable to accurately visualize and quantify complex, mature plant root systems grown under field conditions, they have contributed greatly to increase the precision and efficiency of 2D and 3D spatial and temporal imaging crucial for obtaining information about natural development of RSA in a solid rooting media (reviewed in Danjon and Reubens, 2007; Gregory et al., 2009). Comparative data analysis and integration, especially across controlled environment and field studies is necessary to determine whether QTLs detected by different phenotyping approaches are colocalized along the chromosomes. These regions can be targeted for further investigation to elucidate the genes and molecular mechanisms underlying the trait or phenotype(s) of interest.

The concurrent design of automated or semiautomated image capture systems and software for automated image processing, analysis, and root phenotype quantification (Armengaud et al., 2009; French et al., 2009; Famoso et al., 2010; Clark et al., 2011, 2013) are absolutely essential for simple, precise, and efficient root phenotyping with whole-root system growth platforms. These automated image capture and quantification software systems are also often easily adaptable to an array of low and high-tech growth systems, providing the potential to enhance the throughput and accuracy of root trait measurement from plants grown in a variety of growth systems. Sustained innovation inaccurate, efficient, large-scale, high-throughput root growth and analysis systems, especially those tailored toward more the complex and natural soil and field environments will continue to be essential for future studies on the association and linkage mapping of RSA traits.

## UNDERSTANDING THE GENETIC AND ENVIRONMENTAL CONTROL OF WHOLE SYSTEM ARCHITECTURE

Recent development of new, non-invasive, controlled, root phenotyping techniques and the ability to accurately visualize and quantify RSA paves the way for the further development of higher throughput technologies to assist with linkage and association mapping and mutant analysis. Concurrent advances in the development of informative populations and use of the latest genotyping/sequencing techniques can allow for the faster determination of genes involved in root architectural components and the molecular mechanisms underlying the intrinsic and extrinsic pathways which control root growth and development. 

The next step will be to look at this new root phenotypic data in combination with the well-studied above-ground shoot and yield related traits to determine whether any correlations may be made between root architectural traits and plant performance in different environments. Progress is being made on root–shoot hormone synthesis and signaling pathways (De Kroon et al., 2009; Puig et al., 2012), but the elucidation and integration of the complexes of molecular and hormonal networks that coordinate the developmental regulation with environmental perception and response remains an intriguing opportunity for the plant biology community and a compelling goal for plant breeders who seek new strategies for enhancing crop performance in the face of water and land shortages in the decades to come.

## Conflict of Interest Statement

The authors declare that the research was conducted in the absence of any commercial or financial relationships that could be construed as a potential conflict of interest.
